# Unraveling the stability landscape of mutations in the SARS-CoV-2 receptor-binding domain

**DOI:** 10.1038/s41598-021-88696-5

**Published:** 2021-04-28

**Authors:** Mohamed Raef Smaoui, Hamdi Yahyaoui

**Affiliations:** Computer Science Department, Kuwait University, Kuwait, State of Kuwait

**Keywords:** Computational biology and bioinformatics, Software, Computational biophysics, Computer modelling, Computer science

## Abstract

The interaction between the receptor-binding domain (RBD) of the SARS-CoV-2 spike glycoprotein and the ACE2 enzyme is believed to be the entry point of the virus into various cells in the body, including the lungs, heart, liver, and kidneys. The current focus of several therapeutic design efforts explores attempts at affecting the binding potential between the two proteins to limit the activity of the virus and disease progression. In this work, we analyze the stability of the spike protein under all possible single-point mutations in the RBD and computationally explore mutations that can affect the binding with the ACE2 enzyme. We unravel the mutation landscape of the receptor region and assess the toxicity potential of single and multi-point mutations, generating insights for future vaccine efforts on mutations that might further stabilize the spike protein and increase its infectivity. We developed a tool, called SpikeMutator, to construct full atomic protein structures of the mutant spike proteins and shared a database of 3800 single-point mutant structures. We analyzed the recent 65,000 reported spike sequences across the globe and observed the emergence of stable multi-point mutant structures. Using the landscape, we searched through 7.5 million possible 2-point mutation combinations and report that the (R355D K424E) mutation produces one of the strongest spike proteins that therapeutic efforts should investigate for the sake of developing effective vaccines.

## Introduction

The outbreak of a respiratory illness in Wuhan, China on December 19, 2019 has created a global public emergency and spread a new coronavirus disease (COVID-19)^1,2^. The virus causing COVID-19 was termed SARS-CoV-2^3^, and shares sequence and structural similarity with the Middle East respiratory syndrome coronavirus (MERS-CoV)^4^ and with the severe acute respiratory syndrome coronavirus (SARS-CoV)^5–7^. As of March 30, 2021, the COVID-19 pandemic has infected 128,006,406 people and sadly killed over 2,799,201 worldwide (https://www.worldometers.info/coronavirus/). The pandemic has caused economic shutdown in many countries, fears of a global recession, restricted travel, closure of educational institutions, and mental distress on a global level^8–10^. With the drastic widespread and impact of SARS-CoV-2, many countries are still fighting against the second and third waves of the virus and fear the devastation of future waves^11^.

SARS-CoV-2 contains a lipid envelope bilayer, with attached spike and membrane proteins, surrounding a stranded RNA genome of the virus^12^. Similar to MERS-CoV and SARS-CoV, the spike proteins in SARS-CoV-2 mitigate the attachment and binding of the virus to cell receptors and facilitate the release and entry of the viral genome into host cells^13–15^. The binding to host cells occurs at the receptor-binding domain (RBD)^16^ in the S1 subunit of the spike protein. The mechanical stability of the RBD in SARS-CoV-2 is 50pn greater than in SARS-CoV, which could explain the rapid spread of COVID-19^17^. The SARS-CoV-2 RBD recognizes the angiotensin-converting enzyme 2 (ACE2) located in the lungs, heart, kidney and intestines as its host receptor^2,18^. Impeding the function of spike proteins has been the target of several antibodies, vaccines, and inhibitors^19–22^.

Recent findings have reported the occurrence of sequence mutations in the SARS-CoV-2 spike protein, including some in the RBD^23^. The impact of the mutations on disease progression and biochemical phenotypes in COVID-19 still remains unknown. However, mutations in the spike protein and RBD of SARS-CoV and MERS-CoV are believed to have played a significant evolutionary factor in the transmission of the virus from bats to humans and influenced binding potentials to host receptors^24–26^. Mutations increasing affinity to human receptors are ubiquitous^27,28^, and exploring how mutations in the RBD region of SARS-CoV-2 impact the spike protein would aid in the design of better inhibitors and potential vaccines^29^.

Vaccines targeting the RBD region will have to account for possible natural mutations that could influence the spike protein’s stability and tweak its dynamics with the ACE2 receptor. Mutations altering the RBD conformation have been recently shown to allow SARS-CoV-2 to elude antibody treatments and resist therapy^30^. It is hence imperative to study the possible mutations that could occur in the RBD and the impact they might have on COVID-19 progression, and vaccine interactions. In this study, we computationally explore the mutation landscape of the RBD region and pinpoint mutations expressing strong binding potentials. We developed a tool, called SpikeMutator, to generate all possible single-point spike mutant trimer structures and map their free energies^31,32^ to assess the affect of mutations on structure stability. We analyzed the current isolated spike sequences in the GISAID database^23^ against the energy landscape and found evidence of accumulated mutations increasing the spike’s structural stability. Vaccine efforts targeting spikes will have to account for such mutations as more spike variants start to appear across the globe^33^. To the best of our knowledge, this is the first work that aims to study the mutation landscape of the SARS-CoV-2 RBD region.

## Results and discussion

**Figure 1:**
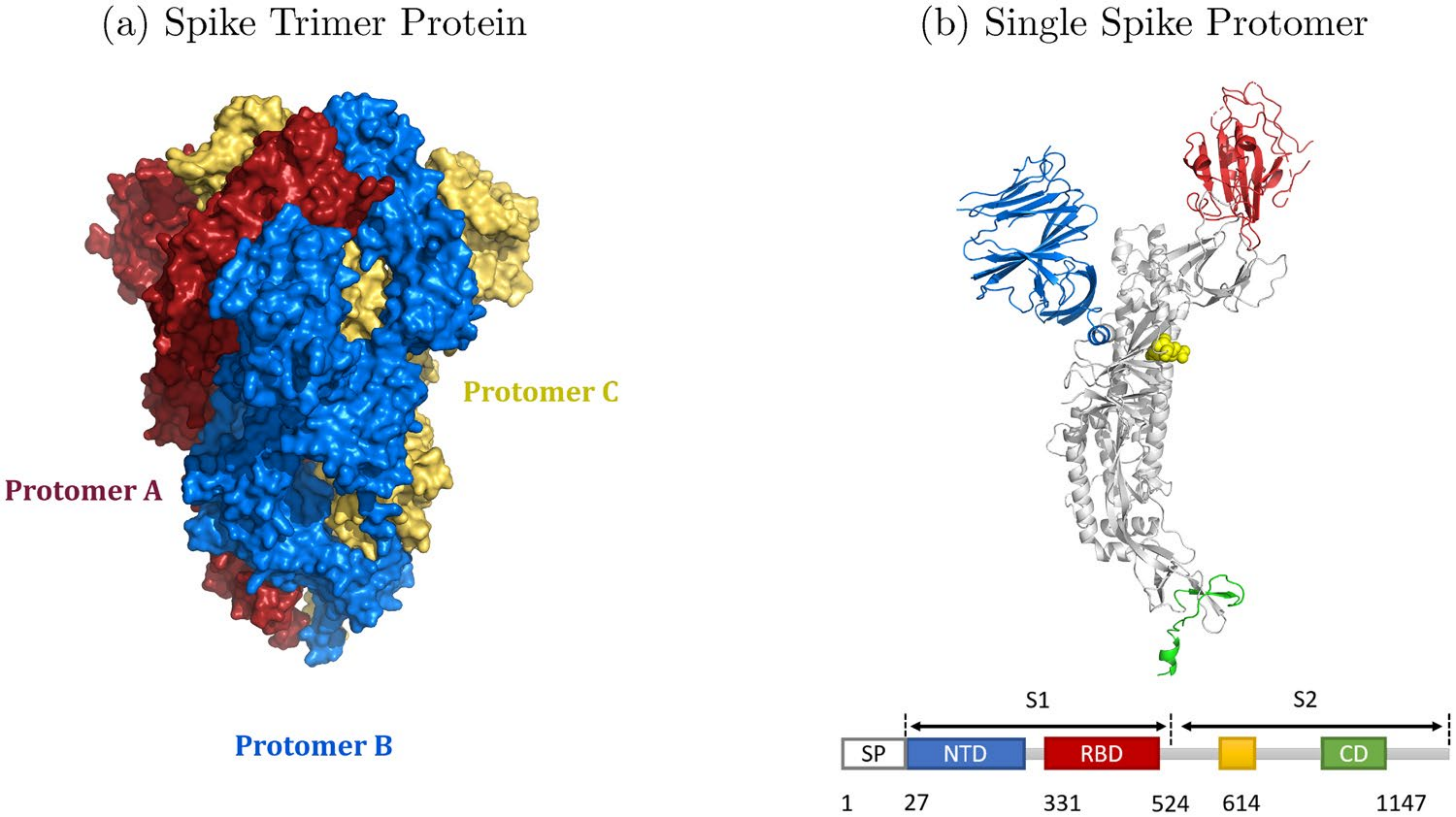
3D Structure of a Spike Protein. (**a**) Crystal structure of the spike protein trimer (PDB ID: 6VXX) composed of three protomers colored red, blue, and yellow, and are all in a closed conformation. (**b**) A single protomer spike in a closed conformation containing a receptor-binding domain highlighted in red, an N-terminal domain highlighted in blue, a connector domain highlighted in green, and position 614 highlighted in yellow.

To analyze the stability of the spike protein against possible mutations that could occur in the RBD region, we developed a tool called *SpikeMutator* to generate a PDB structure of the spike with mutations applied to it. The spike structures we build are based on the cryo-EM SARS-CoV-2 spike glycoprotein reported by Walls et al.^34^. The amino acid sequence of this structure is presented in Table S1. In this structure, the RBD is located between amino acids 331 and 524, inclusive. The spike complex involved in COVID-19 is a trimer structure made up of three spike protomers. Figure 1 presents a schematic image of the spike protein as a single and trimer structure. Three single protomer structures aggregate to form a trimer conformation in Fig. 1a that binds to the ACE2 enzyme at the RBD interface. Each spike protomer contains two functional subunits, S1 and S2. The S1 subunit contains an N-terminal domain (NTD) and a receptor-binding domain (RBD), as highlighted in Fig. 1b. S1 binds to a host receptor and S2 contains the protein’s fusion machinery^35,36^.

**Figure 2:**
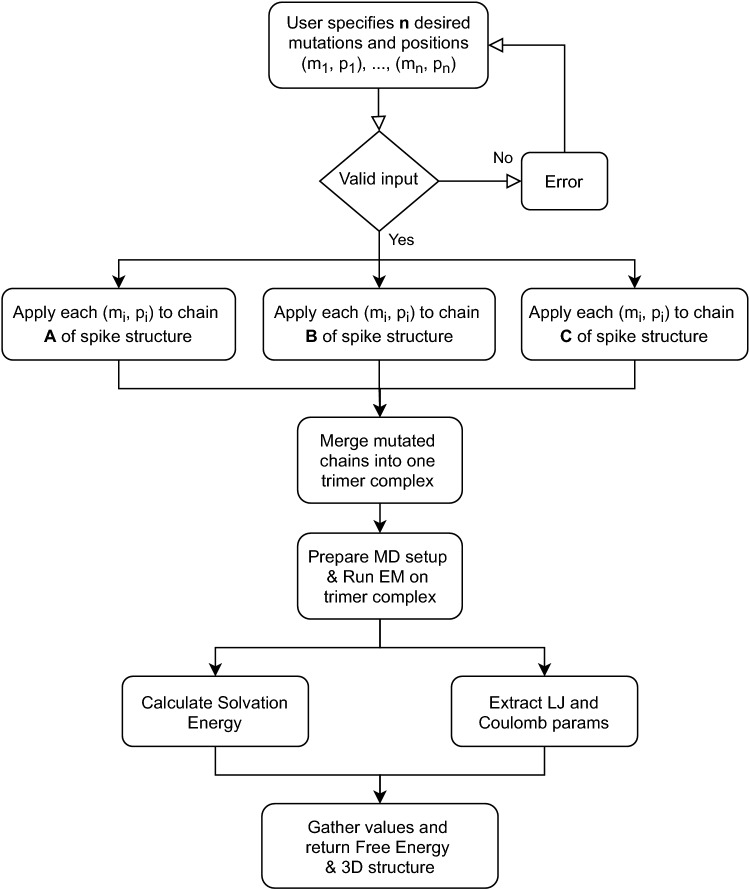
SpikeMutator pipeline. A flowchart describing the methods involved in mutating a SARS-CoV-2 spike protein. Mutations are applied to each trimer in the complex and a resulting atomic structure file is generated along with an output of the resulting free energy.

To generate a spike protein with a set of desired n-point mutations, *SpikeMutator* applies each mutation to all three protomers of the spike complex and runs an all-atom molecular simulation to compute the free energy of a mutant complex using energy terms defined in Eq. (1). Figure 2 presents a flowchart outlining the steps of this tool. The output of the tool is a PDB structure with the desired n-point mutations applied to all three aggregate protomers in the spike complex. The tool supports the construction of the spike complex in both receptor-accessible (open) and receptor-inaccessible (closed) states^36^ and can be used to explore the energetics of the 1up2down and 2up1down spike conformations^37^.

**Figure 3:**
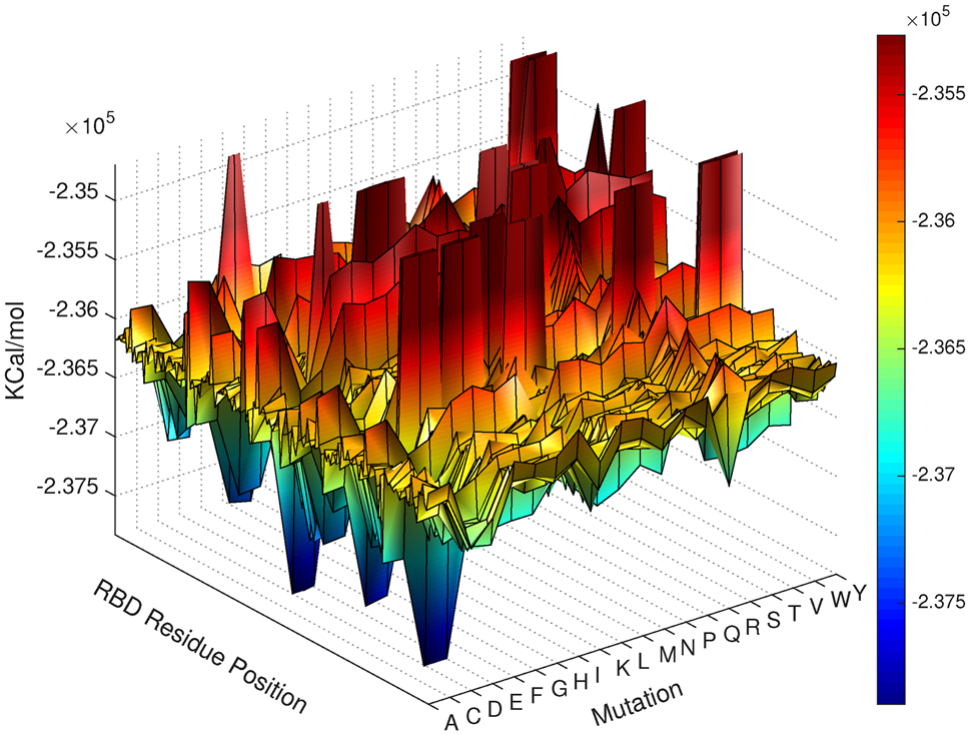
Mutation Landscape of the receptor-binding domain in SARS-CoV-2. Energy values are in kcal/mol and are computed by Eq. (1). Each x,y coordinate represents a mutation x at a position y. The z-values are the free energies of the mutated structures. Lower energies (blue) correspond to increased stability.

**Figure 4:**
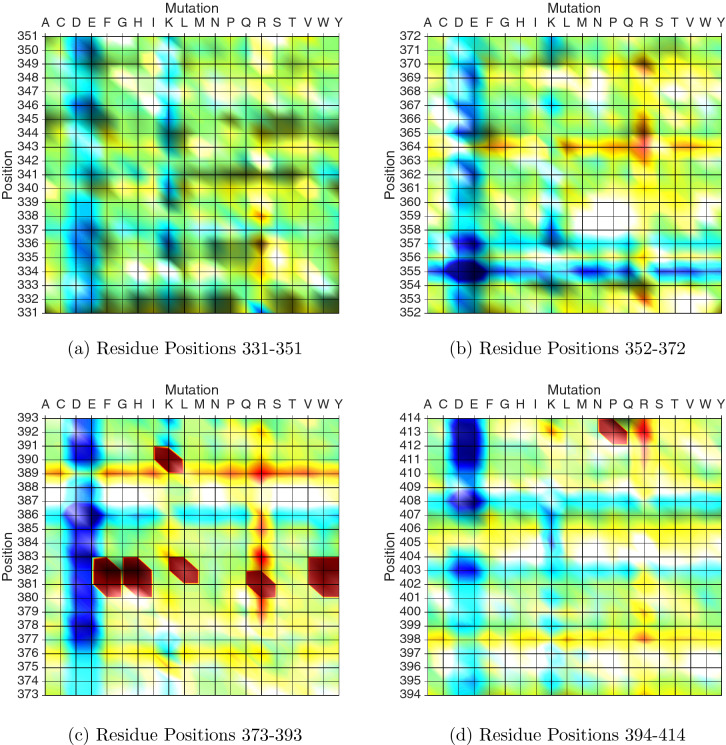

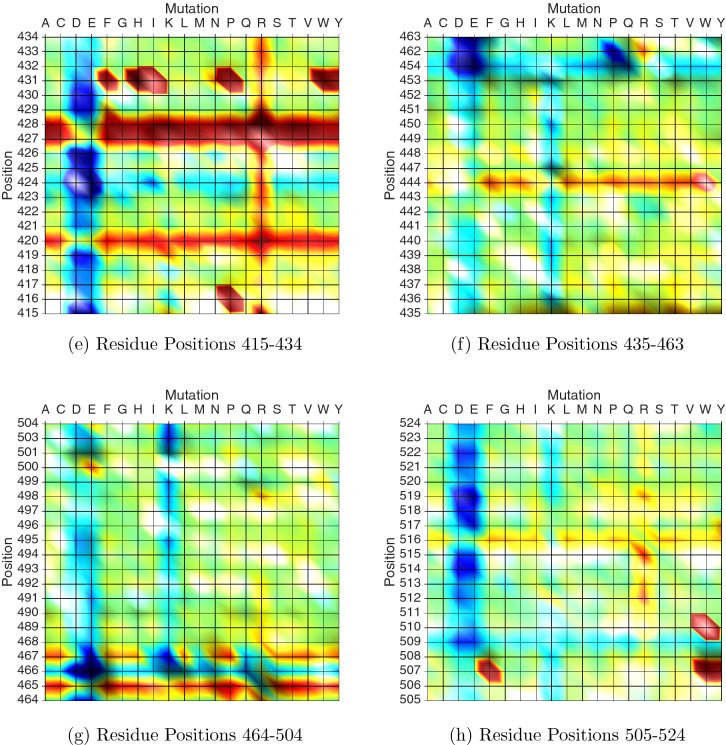
2D Projection of the RBD Mutation Landscape. The introduction of a C or D mutation throughout most positions of the spike consistently suggests an improvement in the overall stability of the protein. Mutations in positions 355, 357, and 408 strikingly result in stable conformations. Positions 364 and 389 appear to be unstable spike regions. 2D Projection of the RBD Mutation Landscape. Positions 420, 427, 428, 444, and 465 appear to be unstable spike regions. On the other hand, positions 454, 462, 466, 501, and others colored in blue can exhibit mutations that stabilize the spike protein.

To study the landscape of potential mutations that can appear in the RBD region, we used *SpikeMutator* to exhaustively mutate each amino acid in the RBD region to the 19 other canonical amino acids and generated a database of the 3D conformations of all possible spike trimer mutants. Every trimer structure contained one mutation that was simultaneously applied to each of the three aggregated spike proteins. The free energies generated by the all-atom simulation runs are reported in Figs. 3 and 4. Figure 3 plots a 3D mutation energy landscape of the receptor-binding domain. One axis in the landscape represents the amino acids positioned at residues 331 through 524, the second axis plots the 20 canonical amino acids, and the third axis captures the free energy of the mutant structure defined by the two other axes. Lower energy values correlate with favorable mutations that stabilize the RBD and can potentially improve binding with the ACE2 enzyme. Higher energy values suggest mutations that increase instability in the domain and potentially alter the binding dynamics with the ACE2 enzyme.

The 3D landscape maps the possible outcomes of single-point mutations and exposes conserved regions and unstable ones. For some amino acid positions, the appearance of a mutation does not impact stability. At other positions, most mutations will cause an increase in destabilizing forces, illustrated in the 3D plot by red curves that create a wall-like barrier. Interestingly, the introduction of some amino acid mutations such as a C or D throughout the spike generally improves structural stability.

Figure 4 provides a 2D projection of the landscape and makes it easier to visualize the energies across different areas of the RBD. We observe that a mutation including a negatively charged, polar and hydrophilic amino acid such as aspartic acid (D) or glutamate (E) would increase the stability of the receptor-binding domain. A positively charged, polar, and hydrophilic amino acid such as arginine (R) introduces some of the most unfavorable mutations in the region 419–434, located relatively inside of the spike trimer and far from the solvent accessible surface. The spike trimer contains an arginine (R) at position 355. The landscape plot suggests that this is an unstable residue position and that any other amino acid mutation at this position would increase the stability of the domain region. Hence, it is highly likely to observe a mutation at this position, which has actually been reported in a sequence from the UK on GISAID^23^. In addition, we found that 15 out of 19 mutations at position 331 and another 15 out of 19 mutations at position 343 increase the instability of the spike complex. Experimentally, it has been shown that disrupting the glycosylation in both of these positions greatly reduce the infectivity of SARS-CoV-2^38^.

Using the 65,000 spike protein sequences collected internationally and made available at GISAID^23^, we found 3405 spike sequences that diverged from the Walls et al.^34^ reference spike sequence. 2491 of those sequences contained missing readings in the receptor-binding domain and were excluded from further analysis. Twenty-one sequences presented indels (insertions and deletions) that might have affected the progression or severity of the disease. A multiple alignment diagram of these sequence is presented in Table S2 of the supplementary material. Countries reporting these sequences with indels are Australia, China, India, Israel, Qatar, UK, and USA. Among these countries, Qatar was the only country to report a spike protein with an insertion resulting in the longest known spike mutant with 196 amino acids. It is not known whether or not this longer sequence contributed to the country’s low COVID-19 mortality rate (0.15%).

The remaining 894 sequences had equal length to the reference spike sequence but exhibited one or more point mutations. We report in Table 1 the top country origins of these sequences and the $\Delta E$ values generated using our mutation landscape and Eq. (2). $\Delta E$ measures the change in free energy between the mutant and non-mutant spike structures. Positive values indicate increase in instability and negative values indicate increase in stability within the RBD. We observe that all the countries in Table 1 report mutations that both stabilize and destabilize the receptor-binding domain, which could explain the varying severity of disease symptoms and health conditions reported in those countries^39^. Table S6 of the supplementary material is an extension of Table 1 and contains the complete list of countries. We report in Table S10 the $\Delta E$ values of the top 10 stabilizing and top 10 destabilizing single-point mutations calculated using three different molecular dynamics forcefields. The results show that the forcefields are in agreement regarding the reported stabilizing mutations. The predicted mutations in the RBD region are increasing the Spike stability. The last column of the table presents an error estimate of the AMBER forcefield we used in comparison with the other forcefields. The error bounds are low, suggesting that the stability outcome of the reported mutations are independent of force-field uncertainties

**Table 1:** Top reporting countries for spike sequences on GISAID.

Reporting origin	Submitted sequences	% of total sequences	Sequences with mutations	Min $\Delta E$	Avg $\Delta E$	Max $\Delta E$
United Kingdom	30,494	47.25	582	− 681	− 268	6966
USA	12,912	20.01	67	− 1094	182	7498
Australia	2052	3.18	28	− 375	267	6966
Spain	1784	2.76	7	− 344	114	600
Netherlands	1582	2.45	30	− 366	− 7	157
India	1563	2.42	26	− 416	− 20	130
Canada	1018	1.58	4	− 497	− 343	64
China	977	1.51	28	− 457	43	593
Belgium	921	1.43	3	32	118	286
Switzerland	726	1.12	4	− 74	− 46	7
Portugal	702	1.09	11	− 37	102	116
Denmark	655	1.01	2	20	51	83
Singapore	638	0.99	3	84	88	91
Japan	627	0.97	2	− 509	− 217	74

Table 2 lists the spike mutations that appear worldwide ranked in order of the number of reporting countries. Mutation V367F was reported in 12 countries and appeared in 51 sequences. Our energy landscape suggests that this mutation is favorable and further stabilizes the receptor-binding domain ($\Delta E < 0$). Other mutations exhibit positive $\Delta E$ values and could lead to different binding potentials with the ACE2 enzyme, affecting the rate of disease, incubation periods and patient symptoms. Table S5 of the supplementary material reports the data for all 894 sequences. Table S7 of the supplementary material presents the point mutations reported in each country as of July 2020.

**Table 2:** Occurrences of spike sequence mutations reported on GISAID.

Mutation	Occurrence	Reporting countries	$\Delta E$ (kcal/mol)	Stabilization rank (%)
V367F	51	12	− 73.64	23.78
P384S	7	6	− 37.3	31.48
A520S	23	6	21.4	52.71
S494P	12	6	83.93	73.35
P384L	21	5	31.53	57.05
V382L	7	5	6966.32	99.82
N439K	422	4	− 417.5	6.92
P463S	8	4	− 43.74	26.74
A348S	8	4	68.61	69.42
A344S	20	4	78.22	71.42
R408I	12	3	− 328.02	10.62
S359N	3	3	− 68.6	24.71
Y508H	4	3	− 33.91	32.22
I468V	6	3	28.91	56.03
H519Q	4	3	66.69	68.92
F490L	4	3	90.57	75.60
A352S	7	3	128.22	83.08
A411S	3	3	143.49	85.17
A435S	3	3	156.87	86.46
G413E	2	2	− 1093.77	0.43
R403K	9	2	− 375.13	8.37
R346K	2	2	− 366.34	8.77
G504D	2	2	− 344.41	9.91
N354K	2	2	− 340.27	10.15
G339D	4	2	− 324.91	10.71
P521S	3	2	− 109.11	20.00
Y505H	2	2	− 104.54	20.28
Q414K	3	2	− 37.51	31.42
A372T	14	2	− 35.55	31.85
I402V	12	2	− 2.36	42.80

Mutation *aPb* denotes the change of amino acid *a* at position *P* to amino acid *b*.

Out of the 894 sequences with mutations, 26 sequences exhibited 2 or more simultaneous point mutations. Table 3 reports these multiple-point mutations. It is interesting to note that all recent 2, 3, and 4-point mutations have a $\Delta E < 0$, suggesting a mutation drive to further stabilize the receptor-binding domain and potentially increase infectivity. The 2-point mutation (V367F G413V) that was reported in Spain appears to have evolved from the widely spread mutated sequence (V367F) reported in 12 countries. No other sequence reported a G413V mutation. Although we do not know the order in which the other mutations have appeared, it is theoretically possible for a sequence to undergo a destabilizing mutation first and then after some time experience a strong stabilizing one that brings the structure to a more overall stable conformation. The sequence with the 3-point mutation (Q506H P507S Y508N) could have undergone its first destabilizing mutation (Q506H $\Delta E > 0$) and then experienced two stabilizing single point mutations (P507S $\Delta E < 0$) and (Y508N $\Delta E < 0$). If this is the case, then other spike sequences that have become mild or less dangerous because of destabilizing mutations could potentially experience future mutations that cause a regain in toxicity potential, and cause a periodic increase and decrease in COVID-19 symptoms.

**Table 3:** Multiple-point mutations in the spike protein reported on GISAID.

m-point mutation	Occurrence	Reporting country	Date	$\Delta {\tilde{E}}$ (kcal/mol)
R509K V510L	1	China	Feb 2020	− 561.68
A411S C432F	1	China	Feb 2020	184.5
G404K D405C	1	India	Mar 2020	− 411.52
D467V I468F	1	Australia	Mar 2020	558.79
N388T S399P	1	Taiwan	Mar 2020	132.0
P491L H519Q	1	Malaysia	Mar 2020	− 118.41
G339D E340K	3	Canada	Apr 2020	− 837.15
V367F G413V	1	Spain	Apr 2020	− 35.96
N439K S494P	1	UK	Jun 2020	− 362.94
P507H Y508N	1	India	Jun 2020	− 182.07
N354D D364Y	1	China	Jan 2020	− 92.46
S399A V407G I410V	1	India	Mar 2020	− 55.45
Q506H P507S Y508N	1	India	Jun 2020	− 118.12
F347I A348P T430N G431S	1	India	Jun 2020	− 114.30

**Table 4:** Prediction scores of top stabilizing 2-point mutations in the receptor-binding domain.

Mutation	SpikeMutator		DynaMut2			PROVEAN	
	$\Delta {{\tilde{E}}}$	$\Delta E$	Error (%)	$\Delta \Delta G$ (kcal/mol)		Score	Function
R355D K424E	− 3418.13	− 3773.73	− 9.4	− 1.49	Stabilize	− 2.42	Affected
R355E K424D	− 3389.30	− 3849.04	− 11.9	− 0.52	Stabilize	− 2.36	Preserved
R355E K424E	− 3357.42	− 3702.01	− 9.3	− 1.32	Stabilize	− 1.93	Preserved
R355D K386D	− 3202.04	− 3456.88	− 7.4	0.01	Neutral	− 2.72	Affected
K386D K424D	− 3182.71	− 3577.51	− 11.0	0.49	Destabilize	− 2.31	Preserved
R355D K386E	− 3168.83	− 3396.34	− 6.7	− 0.24	Stabilize	− 2.28	Affected
K386D K424E	− 3150.83	− 3538.20	− 10.9	0.22	Destabilize	− 1.89	Preserved
K386E K424D	− 3149.50	− 3533.30	− 10.9	0.28	Destabilize	− 1.87	Preserved
R355E K386D	− 3141.33	− 3467.45	− 9.4	− 0.18	Stabilize	− 2.23	Preserved
K386E K424E	− 3117.62	− 3486.90	− 10.6	0.05	Neutral	− 1.44	Preserved

We report in Table S8 of the supplementary material two sequences from China with 6 and 7-point mutations reported in 2019. We have not included these in our analysis as it was strange for that number of mutations to occur early on during the pandemic. A potential vaccine for SARS-CoV-2 or molecular therapeutic, that can inhibit the binding between the receptor-binding domain and the ACE2 enzyme, would need to work on different spike mutants that have started to appear and spread throughout different countries. It is not feasible to create a vaccine tailored to work on each mutant. However, if a vaccine can show good results on one of the most stable mutant structures, then it is possible that it will also show good results on many less stable ones. In light of this, we utilized the energy values of the single-point landscape to generate estimated $\Delta E$ values ($\Delta {{\tilde{E}}}$) for 2-point mutations. Using Eq. (4), we generated $\Delta {{\tilde{E}}}$ values for all possible 7.5 million 2-point mutations. Table 4 reports the top ten most stable 2-point mutations and Table S9 of the supplementary material reports the top 1000. Although not exact, the $\Delta {{\tilde{E}}}$ values given by Eq. (4) are close approximations to the $\Delta E$ values generated from full atomic simulation runs. The margin of error of $\Delta {{\tilde{E}}}$ values was on average 6.5%. This method predicted that the 2-point mutation (R355D K424E) contributes strong structural stability to the spike protein and should be tested against potential vaccines and inhibitors. Other tools in the literature such as DynaMut2^40^, and PROVEAN^41^ have also supported these findings. In Table 4, we report the change in binding affinity induced by mutation ($\Delta \Delta G$) of the top 2-point mutations run on DynaMut2. Positive and negative signs correspond to destabilizing and stabilizing mutations predicted to decrease and increase binding affinity respectively. The PROVEAN tool predicted that the top 2-point mutation (R355D K424E) might have an impact on the biological structure and function of the spike protein.

**Figure 5:**
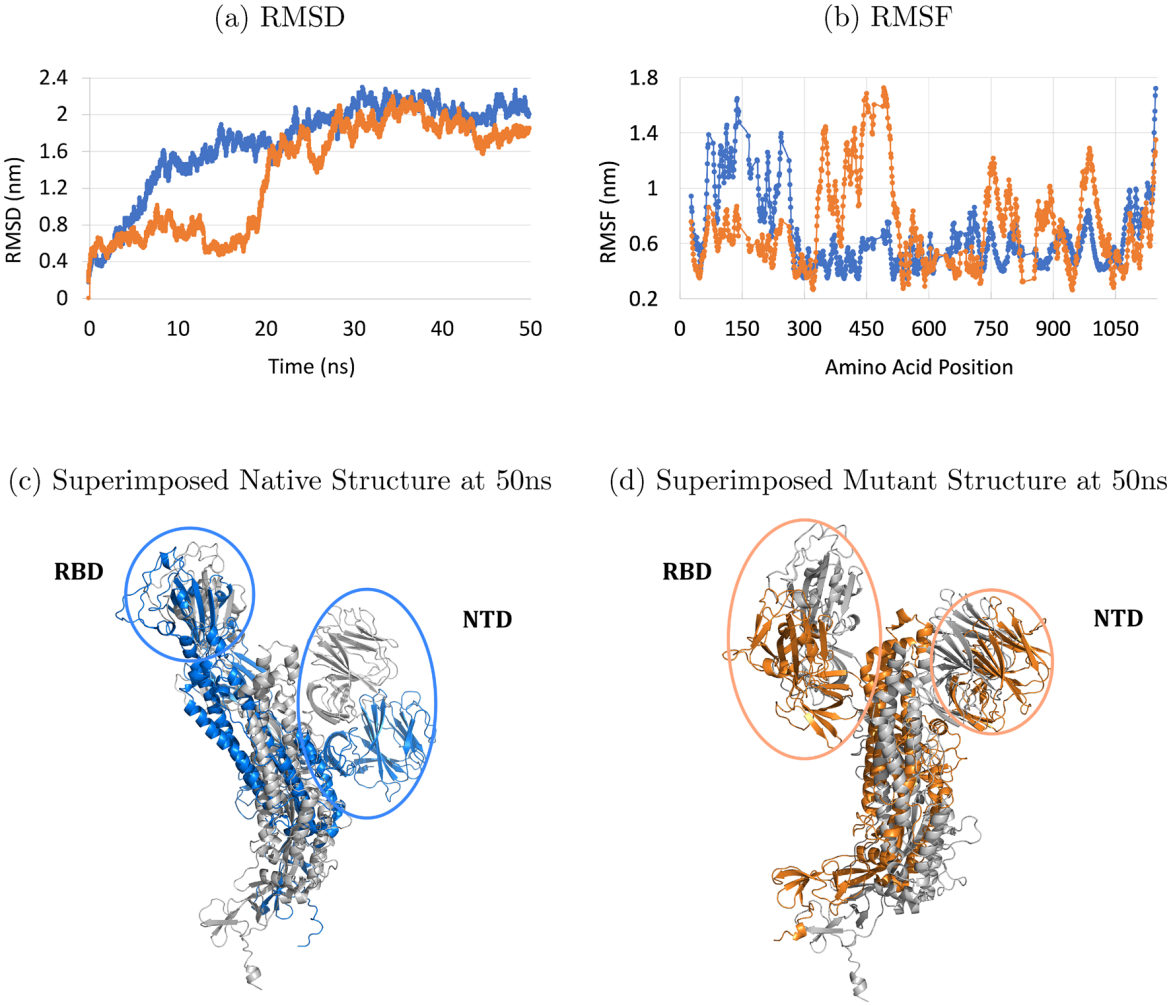
Full 50ns MD Simulation of the non-mutant native spike (blue) and the (R355D K424E) mutant structure (orange). Subfigures (**a**) and (**b**) report the RMSD and RMSF plots, respectively, of the MD simulation run, (**c**) superimposing the non-mutant structure at time 50ns (blue) on the native structure at time 0ns (gray), and (**d**) superimposing the 2-point mutant structure at time 50ns on its starting conformation at time 0ns (gray).

To further explore the effect of the (R355D K424E) mutation on the spike’s structure and stability compared to the non-mutant native type, we ran molecular dynamics simulation of 50 nanoseconds on both the full non-mutant native type and mutant structures. We report in Fig. 5a the RMSD graph of the simulation run. The RMSD graph shows that the mutant structure exhibits a more stable and energetically favorable conformation compared to the non-mutant native type. When we superimposed the resulting structures at time = 50ns onto their initial conformations, we found that the N-terminal domain (NTD) of the mutant structure was more conserved than the NTD in the non-mutant native type. Figure 5c,d shows a schematic of the 2 structures superimposed on their initial starting conformations, plotted in gray. The increase in RMSD values in the mutant graph of Fig. 5a at around time = 20 ns was due to a slight translation shift of the entire RBD region. Figure 5b shows the RMSF plot of the simulation run and captures this change in RBD position. Although the mutation has induced an overall increase in stability, the RBD region’s conformation has been altered. We plot in Figs. S1 and S2 the residue contact map of both the native and mutant structures at time t = 0 ns and t = 50 ns. The results show that the mutant structure is more stable than the native protein. Compared to the native structure, the mutant had lost 15 less contacts, made 4 extra new contacts, and preserved 10 additional contacts. This gain in stability can affect binding to the ACE2 enzyme, and might impose new identification challenges for antibodies. For these reasons, the (R355D K424E) should be tested against potential vaccine candidates.

The 2-point and 3-point mutations that countries have reported are still far from the most stable conformations possible. It appears that the virus still has millions of possible candidate mutations to select from to increase the receptor-binding domain’s stability and potentially become more toxic.

## Methods

The cryo-EM SARS-CoV-2 spike glycoprotein trimer structure (PDB ID 6VXX) was used as a 3D blueprint model on which mutations were performed for “closed” spike conformations and (PDB ID 6VYB) was used as a model for the “open” spike conformations^34^.

### Generating the mutation landscape

Algorithm 1 outlines the process of generating 3D atomic models for all possible single-point mutations in the receptor-binding domain (positions 331-524) and calculating the free energies of each mutant structure. Each of the 194 amino acids in this region was mutated into the 19 other possible canonical amino acids using SCWRL4^42^, producing 3,880 single-point mutation possibilities. Every mutation was applied to each of the three chains in the trimer structure separately and the three structures were subsequently joined and run through an energy minimization step to relax any steric clashes. The result of running Algorithm 1 produced the energy values presented in Figs. 3 and 4.
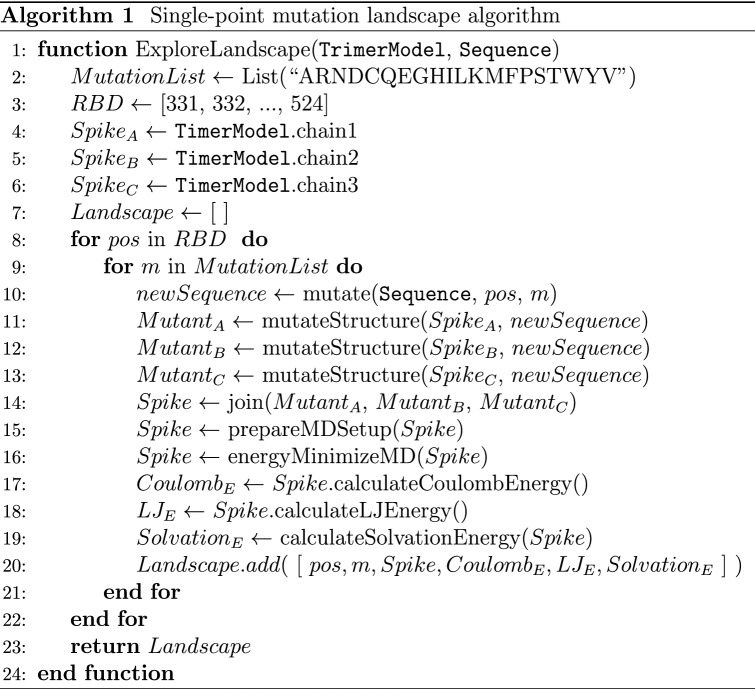


### Computing spike structure energies

The free energy of a protein molecule in a solvent medium is correlated with its structural stability. In general, increased stability promotes better binding potential with other molecules. Lower stability can indicate weaker binding potential. We calculate the free energy $E^K_m$ of a mutant spike structure with a mutation *m* at position *K* by computing the LJ, Coulomb, and solvation energy values of the mutant trimer using Eq. (1),

1 $$\begin{aligned} E^K_m = LJ^K_m + Coul^K_m + S^K_m \end{aligned}$$

where *LJ*$^K_m$ is the Lennard-Jones potential, *Coul*$^K_m$ is the Coulomb energy, and $S$ $^K_m$ measures the solvation energy resulting from the contact of the trimer surface with water molecules for the spike structure with mutation *m* at position *K*. The *LJ* and *Coul* terms measure the electrostatic potential and charges between the atoms of the trimer structure computed after undergoing an energy minimization step to reduce any steric clashes introduced in the mutation phase.

Low free energy values *E* indicate increased stability in the receptor-binding domain, and improved overall stability in the spike protein. Conversely, high *E* values can indicate increased instability in the receptor-binding domain, and lower overall stability in the spike trimer structure.

The *E* values generated in this study are displayed in Fig. 3. The figure describes an energy landscape of all possible single-point mutations in the receptor-binding domain. The amino acid positions of the domain are plotted on the x-axis, the 20 possible mutations on the y-axis, and the *E* value produced by a (position, mutation) pair makes up the z-axis.

Each mutation can be characterized by the difference in energy between its final mutated state and its initial non-mutated state. This change of energy at a position *K* to some amino acid *m* can be captured by Eq. (2),

2 $$\begin{aligned} \Delta E^K_{m} = E^K_m - E^{(0)} \end{aligned}$$

where $E^K_m$ is the free energy of the spike protein with amino acid *m* at position *K* and $E^{(0)}$ is the free energy of the initial non-mutated spike. Negative $\Delta E$ values suggest mutations that increase stability and positive $\Delta E$ values suggest mutations that are potentially destabilizing.

### Solvation energy using dipolar water solvent

The Solvation term is computed using a fast and detailed dipolar water model that solves the dipolar nonlinear Poisson–Boltzmann–Langevin equation using the AQUASOL subroutine^43^. More precisely, the solvation energy in *SpikeMutator* is computed by Eq. (3). To lighten the notation, we omit the indices for the solvation energy term, S.

3 $$\begin{aligned} S &= F_{(p_0, C_{dip})} - F_{(0, 0)} \nonumber \\&\quad-\left( k_B T \frac{ln(1-N_A C_{dip} a ^3)}{N_A C_{dip} a ^3} \right) \int _{solvent} d {\mathbf {r}} \rho _{dip} ({\mathbf {r}}) \end{aligned}$$

where $F_{(p_0, C_{dip})}$ defines the free energy of the system defined at dipoles of moment values $p_0$ and concentration $C_{dip}$, $F_{(0, 0)}$ the free energy of the system with solvent concentration set to zero, $a^3$ is the lattice grid size volume of the solvent, $k_B$ is the Boltzmann constant, *T* temperature in Kelvin, and *r* is the surface definition of the solvent-accessible surface probe. Detailed parameters of the dipolar model setup can be found in the Supplementary Material.

### Energy minimization and molecular dynamics

The process of mutating an amino acid of a structure into another amino acid can often introduce steric clashes with neighbouring residues in its spacial vicinity. After performing a point mutation in each of the three spike chains, *SpikeMutator* joins the three structures back into their initial conformation. Prior to calculating the energy of this new structure, we perform a short energy minimization (EM) run using the GROMACS 2019.3 molecular dynamics package^44^ to relax the structure and remove any severe steric clashes. The setup of the run is as follows: molecules were prepared in a cubic box (with a minimum distance of 35 $\AA$ from any edge of the box to any atom) and neutralized with chloride ions and modeled using the AMBER99SB-ILDN^45^ force field along with the TIP-3P water model. We used a cutoff of 10 $\AA$ for van der Waals and short range electrostatic interactions, and calculated long range electrostatic interactions using a particle mesh Ewald sum^46,47^. 2000 EM steps were performed for every structure using a steepest descent algorithm and the Verlet cut-off scheme. Simulations to generate the RMSD and RMSF plots in Fig. 5 were prepared for a full MD run in both isothermal-isobaric and canonical equilibration ensembles. Twenty five million time steps were used with an integration time step of 2 fs to simulate 50 ns. The residue contact maps of structures undergoing MD have been generated using the GoContactMap server^48^ with the following GO parameters: Sequence distance = 4, Cutoff long = 1.1nm, and Cutoff short = 0.3nm.

### Multiple-point mutations

The number of possible mutations grows exponentially as we consider higher-order point mutations. In the receptor-binding domain, there are 7.5 million possible combinations of 2-point mutations, 9.6 billion possible combinations of 3-point mutations, and 9.2 trillion possible 4-point mutations. Since it is not feasible to efficiently calculate the $\Delta E$ values of these multiple-point mutations, we can estimate the change in energy caused by multiple-point mutations by summing the individual $\Delta E$ values produced by each single-point mutation to produce an estimate change in energy given by Eq. (4),

4 $$\begin{aligned} \Delta {{\tilde{E}}} = \sum _{K~\in ~dom({{\mathcal {M}}})} \Delta E^K_{{{\mathcal {M}}}(K)} \end{aligned}$$

where ${{\mathcal {M}}}$ is a map between spike amino acid positions and mutations, dom(${{\mathcal {M}}}$), returns the positions of the requested mutations, ${{\mathcal {M}}}{(K)}$ returns the desired amino acid mutation at position *K*.

### Supercomputer resources

To perform our simulations, we resorted to the cloud-computing system provided by Amazon Web Services (AWS). Constructing the energy landscape was possible with the utilization of 24 EC2 machines with 16 CPUs each running in parallel for several weeks. The full molecular dynamics simulation of the native and the (R355D K424E) mutant structures required 2 machines with 36 CPUs each. The SpikeMutator algorithm and a copy of the GROMACS 2019.3 molecular dynamics package^44^ was installed on each machine. Generating the 7.5 million possible combinations of 2-point mutations and ranking their energies was also made possible with these machines on AWS.

## Conclusion

We studied in this work the stability of the spike protein under all possible single-point mutations in the receptor-binding domain and explored mutations that can influence structural stability and affect binding with the ACE2 enzyme. We devised a tool, called SpikeMutator, to construct full atomic protein structures of the mutant spike proteins and generated a database of all possible single-point mutant trimer structures. We observed that the sequences isolated from COVID-19 patients exhibited some mutations that both increased and decreased the spike’s structural stability. Out of the 7.5 million possible 2-point mutation combinations, we found that the (R355D K424E) mutation produces one of the most stable spike proteins and should be included in the testing of possible vaccines and molecular inhibitors of SARS-CoV-2.

Our future work will be dedicated to the elaboration of a mutation model that captures the transitions between different spike mutation states in order to detect multiple-point mutations that can cycle between low to high energy states. This would potentially provide some empirical evidence on how mutations can manifest different clinical symptoms. We also aim to explore mutations that appear outside the RBD region, such as the D614G mutation located in position 614 highlighted in Fig. 1b that has become one of the dominant spreading mutations worldwide^49^.

## Supplementary Information

Supplementary Information.

## Data Availability

The 3D conformations of all 3,880 SARS-CoV-2 spike trimer mutants can be accessed at http://spikemutator.com. The *SpikeMutator* program is available for academic use and can be downloaded entirely from the same website.
