# Supplementary material for: Unraveling the stability landscape of mutations in the SARS-CoV-2 receptor-binding domain
Source: Sci Rep. 2021 Apr 28;11:9166. doi: 10.1038/s41598-021-88696-5 (PMC8080587; doi:10.1038/s41598-021-88696-5)
Supplement: Supplementary file 1 — Supplementary Information. [file 41598_2021_88696_MOESM1_ESM.pdf]

Unraveling the stability landscape of mutations in the SARS-CoV-2 receptor-binding domain

M. R. Smaoui* and H. Yahyaoui

Computer Science Department, Kuwait University, Kuwait, State of Kuwait

* *Correspondence to: mohamed.smaoui@ku.edu.kw*

Supplementary Material

Methods

Dipolar water solvent parameters

The Solvation energy is calculated with the following setup: atomic charges and radii assigned with PDB2PQR using CHARMM force field at neutral pH. A grid of 257 points per edge spaced by 1 Å, a temperature of 300K, and a solvent accessible surface with an Rprobe of 1.4 Å. All hydrogen-bonds were optimized. We used a trilinear interpolation protocol for projection of fixed charges on the grid, a lattice grid size for the solvent: a = 2.8 Å, solvent made of dipoles of moment $p_0 = 3.00$D at a concentration of $C_{dip} = 55$M. No salt was added to the solution and small ions were used to equilibrate the system when needed. The electrostatic potential was set to zero at the boundaries, and the stopping criteria for residual was sent to: 1.10^{-6} (when possible). Although using an implicit water model is more computationally expensive, the dipolar model provides more accurate energies and accounts for more realistic atomic interactions.

MD Equilibration

The GROMACS MD equilibration setup was as follows: PME utilization of long-range electrostatics with periodic boundary conditions. Energy Minimization included 2000 iterations of the steepest descent method, followed by 3000 iterations of the conjugate gradient method. Simulations were prepared for a full MD run in both isothermal-isobaric (500 ps) and canonical equilibration (500 ps) ensembles. Temperature and pressure were controlled at 300 K and 1 bar using the velocity rescaling thermostat and the Parrinello-Rahman barostat, respectively. A linear constraint solver was used to keep all bonds at their equilibrium length.

Results

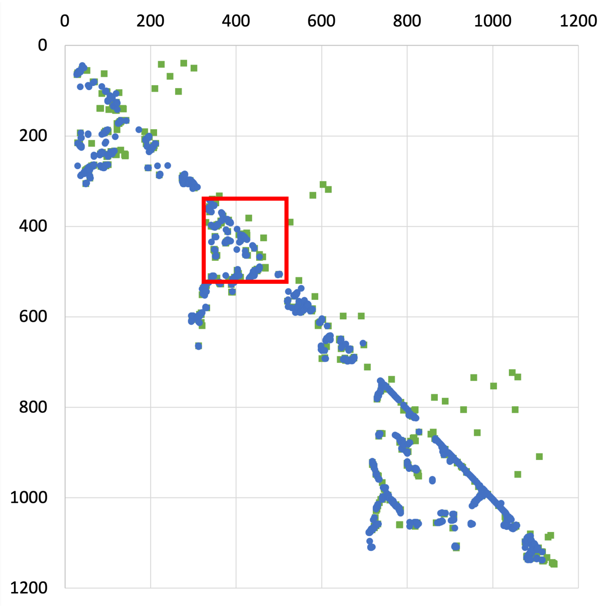

(a) Native t=0ns

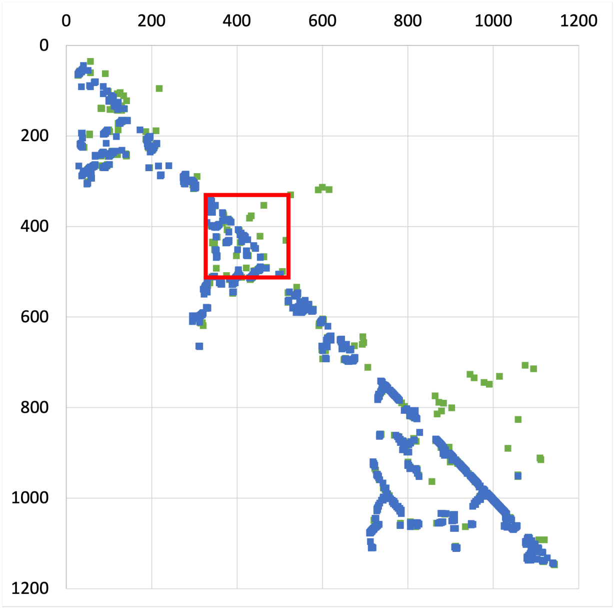

(b) Mutant t=0ns

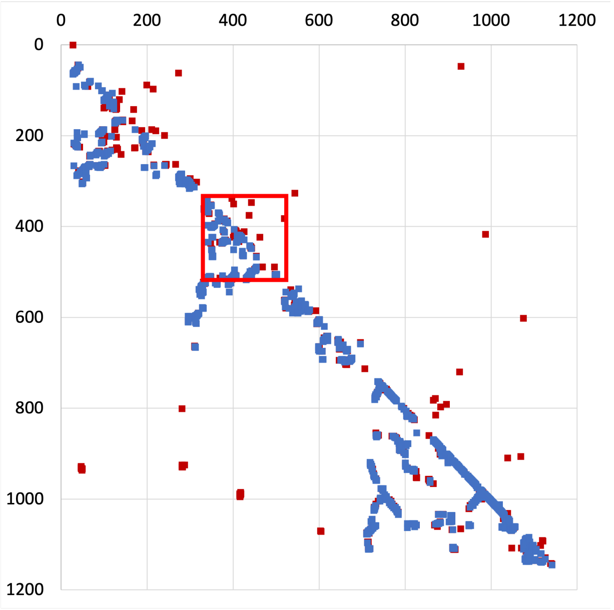

(c) Native t=50ns

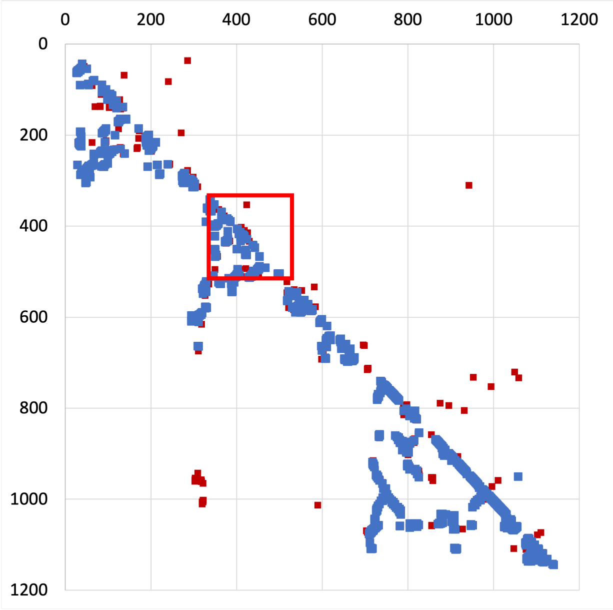

(d) Mutant t=50ns

Figure S1: Residue contact map of the native and top mutant (R355D K424E) structures at t=0ns and t=50ns. The red box outlines the RBD region spanning from residues 331-524. The blue contacts represent conserved contacts between time t=0ns and t=50ns. The green contacts represent contacts that have been lost during the MD run. The red dots represent new contacts that have been formed during the simulation run. The native structure (a) and (c) lost 485 contacts (green), developed 491 new contacts (red), and had 1,449 preserved contacts (blue). The mutant structure (b) and (d) lost 470 contacts (green), developed 495 new contacts (red), and had 1,459 preserved contacts (blue).

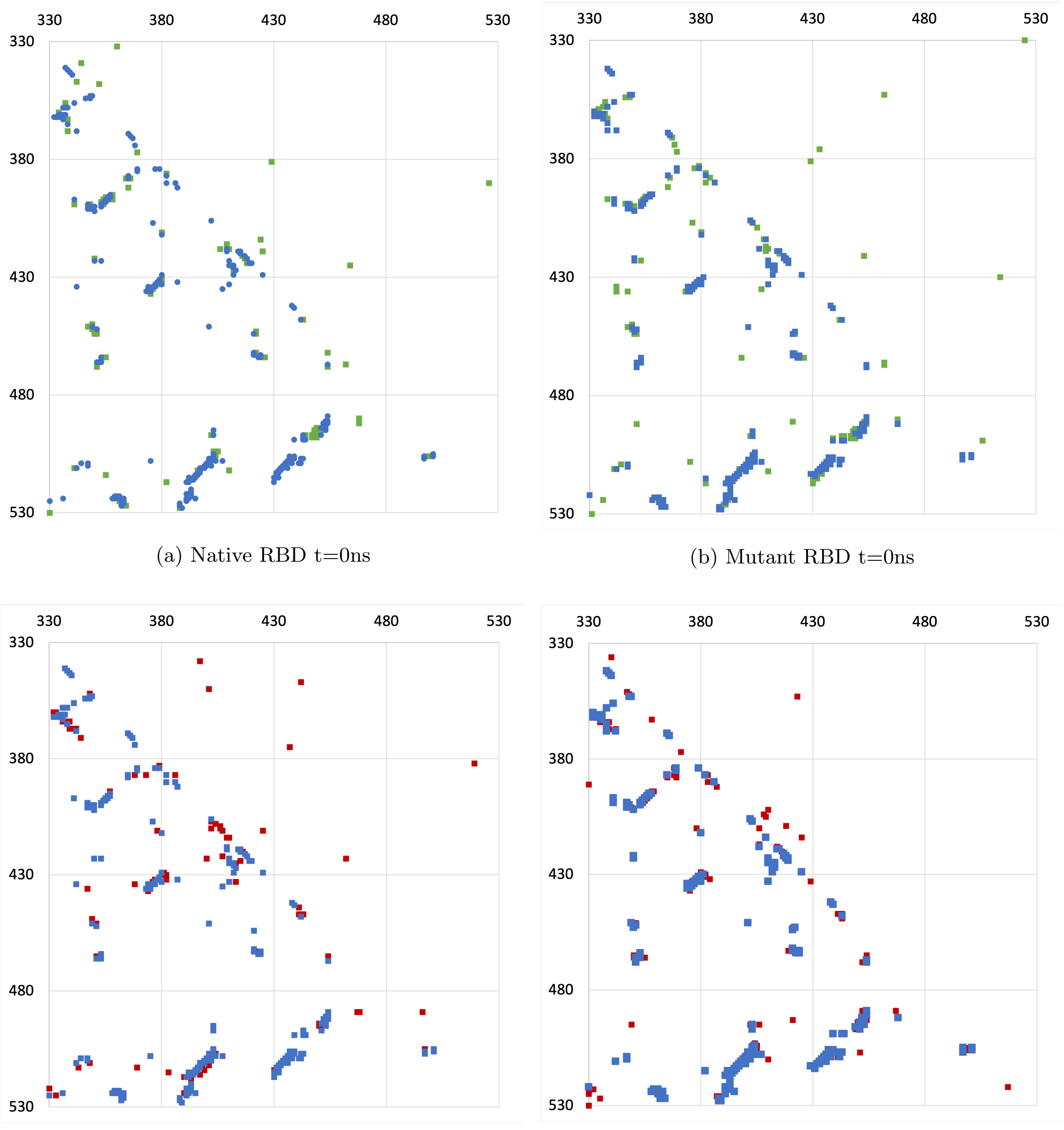

(a) Native RBD t=0ns

(b) Mutant RBD t=0ns

(c) Native RBD t=50ns

(d) Mutant RBD t=50ns

Figure S2: Residue contact map of the native and top mutant (R355D K424E) structures at t=0ns and t=50ns spanning the RBD region. The blue contacts represent conserved contacts between time t=0ns and t=50ns. The green contacts represent contacts that have been lost during the MD run. The red dots represent new contacts that have been formed during the simulation run. Results were generated using the following GO parameters: Sequence distance = 4, Cutoff long = 1.1nm, and Cutoff short = 0.3nm.

Table S1: **SARS-COV-2 Spike Protein Complete Sequence**

```
1------------------20------------------40------------------60-------------------
--------------------------AYTNSFTRGVYYPDKVFRSSVLHSTQDLFLPFFSNVTWFHAIH----------D
81-----------------100-----------------120-----------------140------------------
NPVLPFNDGVYFASTEKSNIIRGWIFGTTLDSKTQSLLIVNNATNVVIKVCEFQFCNDPFLGV-----------------
161----------------180-----------------200-----------------220------------------
----NCTFEYVS-------------FKNLREFVFKNIDGYFKIYSKHTPINLVRDLPQGFSALEPLVDLPIGINITRFQT
241----------------260-----------------280-----------------300------------------
LLALH-----------------AAYYVGYLQPRTFLLKYNENGTITDAVDCALDPLSETKCTLKSFTVEKGIYQTSNFRV
321-------331------340-----------------360-----------------380------------------
QPTESIVRFPNITNLCPFGEVFNATRFASVYAWNRKRISNCVADYSVLYNSASFSTFKCYGVSPTKLNDLCFTNVYADSF
401----------------420-----------------440-----------------460------------------
VIRGDEVRQIAPGQTGKIADYNYKLPDDFTGCVIAWNSNNLDSK--GNYNYLYR-------KPFERDI------------
481----------------500-----------------520-----------------540------------------
--------YFPLQSYGFQPTN-VGYQPYRVVVLSFELLHAPATVCGPKKSTNLVKNKCVNFNFNGLTGTGVLTESNKKFL
561----------------580-----------------600-----------------620------------------
PFQQFGRDIADTTDAVRDPQTLEILDITPCSFGGVSVITPGTNTSNQVAVLYQDVNCTEV--------------------
641----------------660-----------------680-----------------700------------------
NVFQTRAGCLIGAEHVNNSYECDIPIGAGICASYQT------------SQSIIAYTMSLGAENSVAYSNNSIAIPTNFTI
721----------------740-----------------760-----------------780------------------
SVTTEILPVSMTKTSVDCTMYICGDSTECSNLLLQYGSFCTQLNRALTGIAVEQDKNTQEVFAQVKQIYKTPPIKDFGGF
801----------------820-----------------840-----------------860------------------
NFSQILPDPSKPSKRSFIEDLLFNKVT--------------------------KFNGLTVLPPLLTDEMIAQYTSALLAG
881----------------900-----------------920-----------------940------------------
TITSGWTFGAGAALQIPFAMQMAYRFNGIGVTQNVLYENQKLIANQFNSAIGKIQDSLSSTASALGKLQDVVNQNAQALN
961----------------980-----------------1000----------------1020-----------------
TLVKQLSSNFGAISSVLNDILSRLDPPEAEVQIDRLITGRLQSLQTYVTQQLIRAAEIRASANLAATKMSECVLGQSKRV
1041---------------1060----------------1080----------------1100-----------------
DFCGKGYHLMSFPQSAPHGVVFLHVTYVPAQEKNFTTAPAICHDGKAHFPREGVFVSNGTHWFVTQRNFYEPQIITTDNT
1121----------------------1147
FVSGNCDVVIGIVNNTVYDPLQPELDS
```

Complete sequence of the 6VXX PDB structure. The region highlighted in purple is the receptor-binding domain that ranges from residue position 331-524. Missing amino acids in the 3D structure are marked as '-'

Table S2: Multiple Sequence Alignment of the RBD Spikes with indels reported on GISAID

Origin	Sequence
	1--------10--------20--------30--------40-------49
Native	NITNLCPFGEVFNATRFASVYAWNRKRISNCVADYSVLYNSASFSTFKCY
Australia_427104	NITNLCPFGEVFNATRFASVYAWNRKRISNCVADYSVLYNSASFSTFKCY
Australia_480692	NITNLCPFGEVFNATRFASVYAWNRKRISNCVADYSVLYNSASFSTFKCY
Australia_456437	NITNLCPFGEVFNATRFASVYAWNRKRISNCVADYSVLYNSASFSTFKCY
China_410539	NITNLCPFGEVFNASKFASVYAWNRKRISNCVADYSVLYNSTSFSTFKCY
China_463900	NITNLCPFGEVFNATRFASVYAWNRKRISNCVADYSVLYNSASFXXFKCY
China_412860	NITNLCTFGEVFNATTFASVYAWNRKRISNCVADYSVLYNSTSFSTFKCY
India_486673	NITNLCPFGEVFNATRFASVYAWNRKRISNCVADYSVLYNSASFSTFKCY
Israel_447383	NITNLCPFGEVFNATRFASVYAWNRKRISNCVADYSVLYNSASFSTFK-Y
Israel_474996	NITNLCPFGEVFNATRFASVYAWNRKRISNCVADYSVLYNSASFSTFKCY
UK_480330	NITNLCPFGEVFNATRFASVYAWNRKRISNCVADYSVLYNSASFSTFKCY
UK_420942	NITNLCPFGEVFNATRFASVYAWNRKRISNCVADYSVLYNSASFSTFKCY
UK_464255	NITNLCPFGEVFNATRFASVYAWNRKRISNCVADYSVLYNSASFSTFKCY
UK_423566	NITNLCPFGEVFNATRFASVYAWNRKRISNCVADYSVLYNSASFSTFKCY
UK_465705	NITNLCPFGEV-NATRFASVYAWNRKRISNCVADYSVLYNSASFSTFKCY
UK_448231	NITNLCPFGEVFNATRFASVYAWNRKRISNCVADYSVLYNSASFSTFKCY
UK_489687	NITNLCPFGEVFNATRFASVYAWNRKRISNCVADYSVLYNSASFSTFKCY
UK_423651	NITNLCPFGEVFNATRFASVYAWNRKRISNSL----------------CY
UK_423558	NITNLCPFGEVFNATRFASVYAWNR-------------------------
USA_427289	NITNLCPFGEVFNATRFASVYAWNRKRISNCVADYSVLYNSASFSTFKCY
USA_450556	NITNLCPFGEVFNATRFASVYAWNT-------------------------
Qatar_427407	NITNLCPFGEVFNATRFASVYAWNRKRISNCVADYSVLYNSASFSTFKCY
	51-------60--------70--------80--------90-------99
Native	GVSPTKLNDLCFTNVYADSFVIRGDEVRQIAPGQTGKIADYNYKLPDDFT
Australia_427104	GVSPTKLNDLCFTNVYADSFVIRGDEVRQIAPGQTGKIADYNYKLPDDFT
Australia_480692	GVSPTKLNDLCFTNVYADSFVIRGDEVRQIAPGQTGKIADYNYKLPDDFT
Australia_456437	GVSPTKLNDLCFTNVYADSFVIRGDEVRQIAPGQTGKIADYNYKLPDDFT
China_410539	GVSPTKLNDLCFTNVYADSFVVKGDEVRQIAPGQTGVIADYNYKLPDDFT
China_463900	GVSPTKLNDLCFTNVYADSFVIRGDEVRQIAPGQTGKIADYNXX-----X
China_412860	GVSPTKLNDLCFTNVYADSFVVRGDEVRQIAPGQTGRIADYNYKLPDDFT
India_486673	GVSPTKLNDLCFTNVYADSFVIRGDEVRQIAPGQTGKIADYNYKLPDDFT
Israel_447383	GVSPTKLNDLCFTNVYADSFVIRGDEVRQIAPGQTGKIADYNYKLPDDFT
Israel_474996	GVSPTKLNDLCFTNVYADSFVIRGDEVRQIAPG-PGKIADYNYKLPDDFT
UK_480330	GVSPTKLNDLCFTNVYADSFVIRGDEVRQIAPGQTGKIADYNYL--DDFT
UK_420942	GVSPTKLNDLCFTNVYADSFVIRGDEVRQIAPGQTGKIADYNYKLPDDFT
UK_464255	GVSPTKLNDLCFTNVYADSFVIRGDEVRQIAPG-TGKIADYNYKLPDDFT
UK_423566	GVSPTKLNDLCFTNVYADSFVIRGDEVRQIAP--TGKIADYNYKLPDDFT
UK_465705	GVSPTKLNDLCFTNVYADSFVIRGDEVRQIAPGQTGKIADYNYKLPDDFT
UK_448231	GVSPTKLNDLCFTNVYADSFVIRGDEVRQIAPGQTGKIADYNYKLPDDFT
UK_489687	GVSPTKLNDLCFTNVYADSFVIRGDEVRQIAPGQTGKIADYNYKLPDDFT
UK_423651	GVSPTKLNDLCFTNVYADSFVIRGDEVRQIAPGQTGKIADYNYKLPDDFT
UK_423558	---------------------------------KTGKIADYNYKLPDDFT
USA_427289	GVSPTKLNDLCFTNVYADSFVIRGDEVRQIAPGQTGKIADYNYKLPDDFT
USA_450556	------------TNVYADSFVI-GDEVRQIAPGQTGKIADYNYKLPDDFT
Qatar_427407	GVSPTKLNDLCFTNVYADSFVIRGDEVRQIAPGQTGKIADYNYKLPDDFT

Continued on next page

Table S2 – continued from previous page

Origin	Sequence
	101------110-------120-------130-------140-----148
Native	GCVIAWNSNNLDSKVGGNYNYLYRLFRKSNLKPFERDISTEIYQAGSTPC
Australia_427104	GCVIAWNSNNLDSK-------LYRLFRKSNLKPFERDISTE---------
Australia_480692	GCVIAWNSNNLDSKVGGNYNYLYRLFRKSNLKPFERDISTEIYQAG-TPC
Australia_456437	GCVIAWNSNNLDSK--------YRLFRKSNLK-FERDISTEIYQAGSTPC
China_410539	GCVIAWNSVKQDALTGGN--YLYRLFRKSKLKPFERDISTEIYQAGSTPC
China_463900	XXXXXXXXXXLDSKVGGNYNYLYRLFRKSNLKPFERDISTEIYQAGSTPC
China_412860	GCVIAWNSNNLDSKVGGNYNYLYRLFRKSNLKPFERDISTEIYQAGSTPC
India_486673	GCVIAWNSNNLDSKVGGNYNCLYRLFRKSNLKPFERDISTEIY-------
Israel_447383	GCVIAWNSNNLDSKVG-NYNYLYRLFRS-KFE----------------PC
Israel_474996	GCVIAWNSNNLDSKVGGNYNYLYRLFRKSNLKPFERDISTEIYQAGSTPC
UK_480330	GCVIAWNSNNLDSKVGGNYNYLYRLFRKSNLKPFERDISTEIYQAGSTPC
UK_420942	GCVIAWNSNNLDSKVGGNYNYLYRLFRKSNLKPFERDISTEIYQAGSTPC
UK_464255	GCVIAWNSNNLDSKVGGNYNYLYRLFRKSNLKPFERDI-TEIYQA-STPC
UK_423566	GCVIAWNSNNLDSKVGGNYNYLYRLFRKSNLKPFERDISTEIYQAGSTPC
UK_465705	GCVIAWNSNNLDSKVGGNYNYLYRLFRKSNLKPFERDISTEIYQAGSTPC
UK_448231	GCVIAWNSNNLDSKVGGNYNYLYR-FRKSNLKPFERDISTEIYQAGSTPC
UK_489687	GCVIAWNSNNLDSKVGGNYNYLYRLF-----------ISTEIYQAGSTPC
UK_423651	GCVIAWNSNNLDSKVGGNYNYLYRLFRKSNLKPFERDISTEIYQAGSTPC
UK_423558	GCVIAWNSNNLDSKVGGNYNYLYRLFRKSNLKPFERDISTEIYQAGSTPC
USA_427289	GCVIAWNSNNLDSKVGGNYNYLYRLFRKSNLKPFERDISTEIYQAGSTPC
USA_450556	GCVIAWNSNNLDSKVGGNYNYLYRLFRKSNLKPFERDISTEIYQAGSTPC
Qatar_427407	GCVIAWNSNNLDSKVGXXXXXXXXXXXXXXXXXXXXXXXXXXXXXXXXXX
	151------160-------170-------180-------190----
Native	NGVEGFNCYFPLQSYGFQPTNGVGYQPYRVVVLSFELL--HAPATV
Australia_427104	---TPATV
Australia_480692	NGVEGFNCYFPLQSYGFQPTNGVGYQPYRVVVLSFELL--HAPATV
Australia_456437	NGVEGFNCYFPLQSYGFQPTNGVGYQPYRVVVLSFELL--HAPATV
China_410539	NGQVGLNCYYPLERYGFHPTTGVNYQPFRVVVLSFELL--NGPATV
China_463900	NGVEGFNCYFPLQSYGFQXTNGVGYQPYRVVVLSFELL--HAPATV
China_412860	NGVEGFNCYFPLQSYGFHPTNGVGYQPYRVVVLSFELL---APATV
India_486673	--------YFPLQSYGFQPTNGVGYQPYRVVVLSFELL--HAPATV
Israel_447383	---------------GGFQP-NGVGYQPYRV-VLSFELL--HAPATV
Israel_474996	NGVEGFNCYFPLQSYGFQPTNGVGYQPYRVVVLSFELL--HAPATV
UK_480330	NGVEGFNCYFPLQSYGFQPTNGVGYQPYRVVVLSFELL--HAPATV
UK_420942	NGVEGFNCYF-LQSYGFQPTNGVGYQPYRVVVLSFELL--HAPATV
UK_464255	NGVEGFNCYFPLQSYGFQPTNGVGYQPYRVVVLSFELL--HAPATV
UK_423566	NGVEGFNCYFPLQSYGFQPTNGVGYQPYRVVVLSFELL--HAPATV
UK_465705	NGVEGFNCYFPLQSYGFQPTNGVGYQPYRVVVLSFELL--HAPATV
UK_448231	NGVEGFNCYFPLQSYGFQPTNGVGYQPYRVVVLSFELL--HAPATV
UK_489687	NGVEGFNCYFPLQSYGFQPTNGVGYQPYRVVVLSFELL--HAPATV
UK_423651	NGVEGFNCYFPLQSYGFQPTNGVGYQPYRVVVLSFELL--HAPATV
UK_423558	NGVEGFNCYFPLQSYGFQPTNGVGYQPYRVVVLSFELL--HAPATV
USA_427289	NGVEGFNCYFPLQSYGFQPTN-------RVVVLSFELL--HAPATV
USA_450556	NGVEGFNCYFPLQSYGFQPTNGVGYQPYRVVVLSFELL--HAPATV
Qatar_427407	XXXXXXXXXXXXXXXXXXXXXXXXXXXXXXXXXXXXXLXAXXPATV

Table S3: Top Stabilizing 2-point mutations in the receptor-binding domain

Mutation	$\Delta\tilde{E}$ (kcal/mol)	ΔE (kcal/mol)	Discrepancy (kcal/mol)	Error Margin (%)
R355D K424E	-3,418.13	-3,773.73	356	-9.4
R355E K424D	-3,389.30	-3,849.04	460	-11.9
R355E K424E	-3,357.42	-3,702.01	345	-9.3
R355D K386D	-3,202.04	-3,456.88	255	-7.4
K386D K424D	-3,182.71	-3,577.51	395	-11.0
R355D K386E	-3,168.83	-3,396.34	228	-6.7
K386D K424E	-3,150.83	-3,538.20	387	-10.9
K386E K424D	-3,149.50	-3,533.30	384	-10.9
R355E K386D	-3,141.33	-3,467.45	326	-9.4
K386E K424E	-3,117.62	-3,486.90	369	-10.6
R355E K386E	-3,108.12	-3,416.36	308	-9.0
R355D P426D	-2,998.42	-3,160.41	162	-5.1
R355D R466E	-2,995.17	-3,755.49	760	-20.2
R355D F429D	-2,985.36	-2,832.50	-153	5.4
K424D P426D	-2,979.09	-3,226.45	247	-7.7
K424D R466E	-2,975.84	-3,310.17	334	-10.1
K424D F429D	-2,966.03	-2,770.24	-196	7.1
R355D F429E	-2,963.68	-3,068.05	104	-3.4
R355D R466D	-2,950.92	-3,498.29	547	-15.6
K424E P426D	-2,947.21	-3,254.25	307	-9.4
K424D F429E	-2,944.35	-2,811.50	-133	4.7
K424E R466E	-2,943.96	-3,239.15	295	-9.1
R355D G413D	-2,939.45	-2,981.53	42	-1.4
R355E P426D	-2,937.71	-3,098.75	161	-5.2
R355E R466E	-2,934.46	-3,697.82	763	-20.6
K424E F429D	-2,934.15	-2,724.56	-210	7.7
K424D R466D	-2,931.59	-3,215.94	284	-8.8
R355E F429D	-2,924.65	-2,874.42	-50	1.7
G413D K424D	-2,920.12	-3,078.01	158	-5.1
K424E F429E	-2,912.47	-2,720.25	-192	7.1

Table S4: Thermodynamics stability comparison of top reported mutations on GISAID using the I-Mutant and MPTherm-pred tools

Mutation	SpikeMutator ΔE (kcal/mol)	I-Mutant ($\Delta\Delta G$) kcal/mol	MPTherm-pred $\Delta T_m(^{\circ}C)$
V367F	-73.64	-1.67	-1.168
P384S	-37.3	-2.06	-0.362
A520S	21.4	0.03	-2.971
S494P	83.93	-0.16	-8.96
P384L	31.53	-0.23	-1.602
V382L	6966.32	-1.23	1.838
N439K	-417.5	-1.34	-5.683
P463S	-43.74	-1.44	-1.444
A348S	68.61	-1.43	-0.748
A344S	78.22	0.03	-2.422
D614G	445.14	-0.37	-0.642

Table S5: Top reporting countries for Spike sequences on GISAID

Reporting Origin	Submitted Sequences	% of total submission	Sequences with Mutations	% of sequence mutations
United Kingdom	30,494	47.25	582	65.10
USA	12,912	20.01	67	7.49
Australia	2,052	3.18	28	3.13
Spain	1,784	2.76	7	0.78
Netherlands	1,582	2.45	30	3.36
India	1,563	2.42	26	2.91
Canada	1,018	1.58	4	0.45
China	977	1.51	28	3.13
Belgium	921	1.43	3	0.34
Switzerland	726	1.12	4	0.45
Portugal	702	1.09	11	1.23
Denmark	655	1.01	2	0.22
Singapore	638	0.99	3	0.34
Japan	627	0.97	2	0.22
Iceland	595	0.92	3	0.34
Brazil	580	0.90	7	0.78
Sweden	532	0.82	26	2.91
France	406	0.63	8	0.89
Austria	404	0.63	0	0.00
South Africa	335	0.52	7	0.78
Germany	321	0.50	4	0.45
Israel	278	0.43	5	0.56
Luxembourg	270	0.42	2	0.22
Finland	265	0.41	1	0.11
Russia	252	0.39	1	0.11
Bangladesh	228	0.35	4	0.45
Thailand	228	0.35	0	0.00
New Zealand	206	0.32	0	0.00
Italy	182	0.28	0	0.00
Chile	166	0.26	0	0.00
Oman	160	0.25	1	0.11
Democratic Republic of the Congo	150	0.23	1	0.11
Saudi Arabia	142	0.22	1	0.11
Turkey	138	0.21	3	0.34
Colombia	116	0.18	0	0.00
Taiwan	113	0.18	2	0.22
Greece	111	0.17	1	0.11
Romania	111	0.17	4	0.45
Senegal	111	0.17	2	0.22
Egypt	104	0.16	3	0.34
Poland	82	0.13	2	0.22
South Korea	78	0.12	0	0.00
Kenya	74	0.11	0	0.00
Norway	62	0.10	2	0.22
Hungary	61	0.09	0	0.00
Vietnam	56	0.09	0	0.00
Kazakhstan	53	0.08	0	0.00
Latvia	53	0.08	2	0.22
Malaysia	50	0.08	2	0.22
Czech Republic	49	0.08	0	0.00

Continued on next page

Table S5 – continued from previous page

Reporting Origin	Submitted Sequences	% of total submission	Sequences with Mutations	% of sequence mutations
Ecuador	49	0.08	0	0.00
Morocco	48	0.07	1	0.11
Costa Rica	45	0.07	0	0.00
Uruguay	37	0.06	0	0.00
Argentina	36	0.06	0	0.00
Bahrain	35	0.05	0	0.00
Peru	35	0.05	0	0.00
Nigeria	33	0.05	0	0.00
United Arab Emirates	32	0.05	0	0.00
Bosnia and Herzegovina	31	0.05	1	0.11
Jordan	28	0.04	0	0.00
Mexico	28	0.04	0	0.00
Philippines	23	0.04	0	0.00
Croatia	22	0.03	0	0.00
Georgia	21	0.03	0	0.00
Mali	21	0.03	0	0.00
Estonia	19	0.03	0	0.00
Uganda	19	0.03	0	0.00
Gambia	17	0.03	0	0.00
Indonesia	16	0.02	1	0.11
Bulgaria	15	0.02	0	0.00
Ireland	15	0.02	0	0.00
Serbia	15	0.02	0	0.00
Qatar	14	0.02	0	0.00
Benin	12	0.02	0	0.00
Timor-Leste	9	0.01	0	0.00
Cyprus	8	0.01	0	0.00
Jamaica	8	0.01	0	0.00
Kuwait	8	0.01	0	0.00
Pakistan	8	0.01	0	0.00
Tunisia	8	0.01	0	0.00
Iran	6	0.01	0	0.00
Lebanon	6	0.01	0	0.00
Brunei	5	0.01	0	0.00
Slovenia	5	0.01	0	0.00
Sri Lanka	5	0.01	0	0.00
Slovakia	4	0.01	0	0.00
Algeria	3	0.00	0	0.00
Belarus	3	0.00	0	0.00
Ghana	3	0.00	0	0.00
Guam	3	0.00	0	0.00
Lithuania	3	0.00	0	0.00
Venezuela	3	0.00	0	0.00
Cambodia	1	0.00	0	0.00
Mongolia	1	0.00	0	0.00
Myanmar	1	0.00	0	0.00
Nepal	1	0.00	0	0.00
Panama	1	0.00	0	0.00

Table S6: Occurrences of Spike sequence mutations reported on GISAID

**Mutation**	**Occurrence**	**Reporting Countries**	**ΔE (kcal/mol)**
V367F	51	12	-73.64
P384S	7	6	-37.3
A520S	23	6	21.4
S494P	12	6	83.93
P384L	21	5	31.53
V382L	7	5	6966.32
N439K	422	4	-417.5
P463S	8	4	-43.74
A348S	8	4	68.61
A344S	20	4	78.22
R408I	12	3	-328.02
S359N	3	3	-68.6
Y508H	4	3	-33.91
I468V	6	3	28.91
H519Q	4	3	66.69
F490L	4	3	90.57
A352S	7	3	128.22
A411S	3	3	143.49
A435S	3	3	156.87
G413E	2	2	-1093.77
R403K	9	2	-375.13
R346K	2	2	-366.34
G504D	2	2	-344.41
N354K	2	2	-340.27
G339D	4	2	-324.91
P521S	3	2	-109.11
Y505H	2	2	-104.54
Q414K	3	2	-37.51
A372T	14	2	-35.55
I402V	12	2	-2.36
L441I	2	2	1.95
S494L	2	2	6.51
N501Y	15	2	7.55
T385I	2	2	23.52
I468T	3	2	23.94
F490S	8	2	33.74
K417N	4	2	38.45
Y453F	7	2	53.12
A520V	3	2	63.54
V341I	27	2	69.53
I410V	4	2	80.14
L452R	2	2	82.53
S399P	2	2	83.4
P463L	2	2	92.88
N370S	11	2	115.69
A344T	2	2	136.36
N501T	2	2	156.99
P521R	5	2	186.19
T376I	3	2	236.71
E516Q	2	2	285.6
Q414R	9	2	530.42

Continued on next page

Table S6 – continued from previous page

Mutation	Occurrence	Reporting Countries	ΔE (kcal/mol)
K417R	12	2	537.18
R355S	1	1	-681.42
V382E	1	1	-666.58
R357K	1	1	-509.14
E340K	3	1	-496.58
N450K	1	1	-493.48
N440K	1	1	-459.5
R509K	1	1	-457.18
Q409E	1	1	-452.78
G404K	1	1	-415.89
N354D	2	1	-334.33
H519K	2	1	-294.47
P337S	2	1	-213.43
R403S	1	1	-209.62
R403T	1	1	-122.92
P491L	1	1	-119.24
N501D	1	1	-111.68
V510L	1	1	-89.5
P499S	1	1	-88.08
T430N	1	1	-81.63
V433A	1	1	-78.65
Q493Y	1	1	-75.92
P507H	1	1	-66.01
Y508N	2	1	-62.54
R346T	12	1	-54.68
P507S	1	1	-54.16
V401L	1	1	-52.2
V503F	1	1	-47.54
G413A	1	1	-39.05
S399A	1	1	-35.83
P521L	1	1	-32.33
T430A	1	1	-32.02
Y495N	1	1	-29.27
T415S	1	1	-27.23
G431S	1	1	-22.07
S514Y	1	1	-18.3
V367I	8	1	-16.07
I402L	2	1	-14.88
V362F	1	1	-14.41
A352V	1	1	-14.19
V407G	1	1	-13.59
F490Y	1	1	-13.03
F347I	1	1	-12.87
A372S	1	1	-12.03
I434M	1	1	-11.62
F377L	6	1	-7.38
G496C	1	1	-2.98
F338L	3	1	0.04
F342C	1	1	0.11
L517F	1	1	10.09
V503I	1	1	10.81
T385A	2	1	13.35

Continued on next page

Table S6 – continued from previous page

Mutation	Occurrence	Reporting Countries	ΔE (kcal/mol)
L368P	1	1	14.62
V407R	1	1	17.12
Q498Y	1	1	18.85
T415I	1	1	19.69
K378N	2	1	19.93
K356N	1	1	23.31
G504V	1	1	24.85
P499H	1	1	26.36
T500I	1	1	26.36
Q493L	3	1	27.41
H519N	10	1	34.95
V395I	1	1	36.34
S349F	1	1	36.81
S373L	1	1	36.82
A348P	1	1	39.36
N440H	1	1	42.91
Q506H	1	1	43.23
I468F	1	1	44.27
C432F	1	1	47.81
S514F	1	1	47.83
T345S	1	1	48.25
I358L	1	1	51.09
C379F	1	1	53.98
S443A	1	1	55.68
N501H	1	1	57.6
Y449N	1	1	61
N388T	1	1	63.12
Q493R	1	1	64.23
A348T	1	1	64.82
W353S	1	1	65.11
I418V	1	1	66.85
L425F	1	1	67.58
N394Y	1	1	74.38
Q498H	12	1	75.13
N354S	7	1	79.18
N501S	1	1	95.66
S494R	1	1	107.27
L390P	1	1	108.5
Q414P	1	1	108.7
Y449F	1	1	112.84
E406Q	1	1	114.15
D405C	1	1	130
L518I	3	1	144.1
W353L	1	1	148.34
G413V	1	1	152.48
T393P	1	1	168.48
H519P	1	1	190.07
P491R	1	1	210.01
D364Y	1	1	290.61
E516V	1	1	334.35
K356R	1	1	341.04
C336R	1	1	409.81

Continued on next page

Table S6 – continued from previous page

Mutation	Occurrence	Reporting Countries	ΔE (kcal/mol)
W353R	1	1	520.76
P463R	1	1	551.01
D467V	1	1	571.31
K378R	1	1	593.12
K444R	1	1	600.2
D428G	1	1	820.96
D427Y	1	1	910.04
G381R	1	1	7498.18

Table S7: Recorded Mutations in each reporting coutry

Country	Mutation(s)
Australia	A520S
	D467V
	F490L
	G504D
	H519Q
	I468F
	I468T
	N501Y
	P491R
	R403K
	V367F
	V382L
	W353R
Bangladesh	E516Q
	L518I
Belgium	E516Q
	P384L
	S373L
Bosnia and Herzegovina	T430A
Brazil	A348S
	A520S
	I434M
	T376I
	V382L
Canada	A520V
	E340K
	G339D
China	A344T
	A372T
	A411S
	C432F
	D364Y
	F490Y
	H519K
	H519N
	H519P
	I402V
	K378R
	K417R
	L425F
	L441I
	N354D
	N439K
	N440H
	N501D
	Q409E
	Q493Y
	Q498H
	Q498Y
	R346T

Continued on next page

Table S7 – continued from previous page

Country	Mutation(s)
	R403T
	R509K
	S349F
	S359N
	S443A
	S494R
	V367F
	V510L
	Y449F
	Y505H
Democratic Republic of the Congo	I402V
Denmark	L452R
	T415I
Egypt	P499H
	R408I
Finland	A435S
France	C379F
	V367F
	V382E
	Y508H
Germany	K417N
	N501T
	V367F
Greece	T393P
Iceland	K356N
	V367F
	W353L
India	A344S
	A348P
	A348S
	A520S
	D405C
	F347I
	F490S
	G404K
	G431S
	G504V
	I402L
	I410V
	L368P
	P507H
	P507S
	P521S
	Q506H
	R408I
	S399A
	S494P
	T430N
	V367F
	V407G

Continued on next page

Table S7 – continued from previous page

Country	Mutation(s)
	V407R
	V433A
	Y508N
Indonesia	A352S
Israel	A435S
	K417R
	Q414K
	T415S
Japan	N394Y
	R357K
Latvia	N440K
	P384S
Luxembourg	P463L
	Y495N
Malaysia	H519Q
	P491L
	Y449N
Morocco	C336R
Netherlands	A352S
	A435S
	A520S
	L452R
	N354K
	N501T
	P384S
	R346K
	V367F
	Y453F
	Y508H
Norway	G339D
	T385I
Oman	S514Y
Poland	P337S
Portugal	N370S
	P384S
Romania	N439K
	P521R
	Q414P
Russia	P384L
Saudi Arabia	A344S
Senegal	V382L
Singapore	F490L
	S494P
South Africa	G413E
	G496C
	I468V
	P384L
	P384S

Continued on next page

Table S7 – continued from previous page

Country	Mutation(s)
	Y453F
Spain	G413V
	G504D
	K444R
	L517F
	P463S
	Q414R
	S494P
	V367F
Sweden	Q414K
	S494P
	V341I
	Y508H
Switzerland	P463S
	S494L
	V367F
Taiwan	N388T
	S399P
	V367F
Turkey	I468V
United Kingdom	A344S
	A344T
	A348S
	A352S
	A352V
	A372S
	A372T
	A411S
	A520S
	A520V
	D427Y
	E406Q
	E516V
	F338L
	F377L
	F490S
	G413A
	I358L
	I410V
	I468T
	I468V
	K356R
	K378N
	K417N
	N354S
	N370S
	N439K
	N450K
	N501H
	N501S
	P384L

Continued on next page

Table S7 – continued from previous page

Country	Mutation(s)
	P384S
	P463L
	P463S
	P499S
	P521L
	P521S
	Q414R
	Q493R
	R355S
	R403S
	R408I
	S359N
	S399P
	S494L
	S494P
	S514F
	T500I
	V341I
	V362F
	V367F
	V367I
	V382L
	V503I
	W353S
	Y505H
USA	A344S
	A348S
	A348T
	A411S
	A520S
	D428G
	F342C
	F490L
	G381R
	G413E
	H519Q
	I418V
	L390P
	L441I
	N354K
	N439K
	N501Y
	P384L
	P384S
	P463R
	P463S
	P521R
	Q493L
	R346K
	R403K
	S359N
	S494P

Continued on next page

Table S7 – continued from previous page

Country	Mutation(s)
	T345S
	T376I
	T385A
	T385I
	V367F
	V382L
	V395I
	V401L
	V503F

Table S8: Multiple-point mutations in the Spike protein reported on GISAID

m-point Mutation	Occurrence	Reporting Country	Date	$\Delta\tilde{E}$ (kcal/mol)
R346T A372T I402V K417R Q498H H519N	10	China	2019	15
R346T A372T I402V K417R Q498H H519N L425F	1	China	2019	16

Table S9: Top 1000 Stabilizing 2-point mutations in the receptor-binding domain

Mutation	$\Delta\tilde{E}$
R355D K424E	-3,418.13
R355E K424D	-3,389.30
R355E K424E	-3,357.42
R355D K386D	-3,202.04
K386D K424D	-3,182.71
R355D K386E	-3,168.83
K386D K424E	-3,150.83
K386E K424D	-3,149.50
R355E K386D	-3,141.33
K386E K424E	-3,117.62
R355E K386E	-3,108.12
R355D P426D	-2,998.42
R355D R466E	-2,995.17
R355D F429D	-2,985.36
K424D P426D	-2,979.09
K424D R466E	-2,975.84
K424D F429D	-2,966.03
R355D F429E	-2,963.68
R355D R466D	-2,950.92
K424E P426D	-2,947.21
K424D F429E	-2,944.35
K424E R466E	-2,943.96
R355D G413D	-2,939.45
R355E P426D	-2,937.71
R355E R466E	-2,934.46
K424E F429D	-2,934.15
K424D R466D	-2,931.59
R355E F429D	-2,924.65
G413D K424D	-2,920.12
K424E F429E	-2,912.47
R355D G413E	-2,910.31
R355E F429E	-2,902.97
K424E R466D	-2,899.71
G413E K424D	-2,890.98
R355E R466D	-2,890.21
G413D K424E	-2,888.24
R355E G413D	-2,878.74
R355D K462P	-2,866.94
R355D T415D	-2,860.35
G413E K424E	-2,859.10
R355E G413E	-2,849.60
K424D K462P	-2,847.61
T415D K424D	-2,841.02
R355D R408D	-2,832.10
R355D R408E	-2,816.42
K424E K462P	-2,815.73
R408D K424D	-2,812.77
R355D R454D	-2,809.84
T415D K424E	-2,809.14
R355E K462P	-2,806.23
R355E T415D	-2,799.64

Continued on next page

Table S9 – continued from previous page

Mutation	$\Delta\tilde{E}$
R408E K424D	-2,797.09
R355D R454E	-2,792.38
K424D R454D	-2,790.51
R355D D467E	-2,788.41
R355D P412E	-2,787.43
R408D K424E	-2,780.89
K424D R454E	-2,773.05
R355E R408D	-2,771.39
K424D D467E	-2,769.08
P412E K424D	-2,768.10
R408E K424E	-2,765.21
K424E R454D	-2,758.63
R355E R408E	-2,755.71
R355E R454D	-2,749.13
K424E R454E	-2,741.17
K424E D467E	-2,737.20
P412E K424E	-2,736.22
R355D H519E	-2,734.18
R355E R454E	-2,731.67
K386D P426D	-2,731.12
K386D R466E	-2,727.87
R355E D467E	-2,727.70
R355E P412E	-2,726.72
K386D F429D	-2,718.06
R355D P426E	-2,716.27
K424D H519E	-2,714.85
R355D T415E	-2,710.71
R355D H519D	-2,707.69
K386E P426D	-2,697.91
K424D P426E	-2,696.94
K386D F429E	-2,696.38
K386E R466E	-2,694.66
T415E K424D	-2,691.38
K424D H519D	-2,688.36
R355D P412D	-2,685.16
K386E F429D	-2,684.85
K386D R466D	-2,683.62
K424E H519E	-2,682.97
R355D S383E	-2,679.97
R355E H519E	-2,673.47
K386D G413D	-2,672.15
R355D R357E	-2,671.48
P412D K424D	-2,665.83
K424E P426E	-2,665.06
K386E F429E	-2,663.17
S383E K424D	-2,660.64
T415E K424E	-2,659.50
R355D S383D	-2,657.67
K424E H519D	-2,656.48
R355E P426E	-2,655.56
R357E K424D	-2,652.15
K386E R466D	-2,650.41

Continued on next page

Table S9 – continued from previous page

Mutation	$\Delta\tilde{E}$
R355E T415E	-2,650.00
R355E H519D	-2,646.98
R355D A419D	-2,646.38
R355D D467K	-2,644.53
K386D G413E	-2,643.01
K386E G413D	-2,638.94
S383D K424D	-2,638.34
R355D K378D	-2,637.77
R355D T430D	-2,635.24
R355D P463D	-2,634.39
P412D K424E	-2,633.95
R355D S514D	-2,631.01
R355D A411D	-2,630.67
S383E K424E	-2,628.76
R355D C391D	-2,627.84
A419D K424D	-2,627.05
R355D K378E	-2,625.86
K424D D467K	-2,625.20
R355E P412D	-2,624.45
R357E K424E	-2,620.27
R355E S383E	-2,619.26
K378D K424D	-2,618.44
K424D T430D	-2,615.91
K424D P463D	-2,615.06
K424D S514D	-2,611.68
A411D K424D	-2,611.34
R355E R357E	-2,610.77
K386E G413E	-2,609.80
R355D R403D	-2,609.60
C391D K424D	-2,608.51
K378E K424D	-2,606.53
S383D K424E	-2,606.46
K386D K462P	-2,599.64
R355D A411E	-2,598.37
R355E S383D	-2,596.96
A419D K424E	-2,595.17
K424E D467K	-2,593.32
K386D T415D	-2,593.05
R355D T385E	-2,592.69
R403D K424D	-2,590.27
R355D R357D	-2,589.89
R355D G381D	-2,587.40
K378D K424E	-2,586.56
R355E A419D	-2,585.67
K424E T430D	-2,584.03
R355E D467K	-2,583.82
K424E P463D	-2,583.18
K424E S514D	-2,579.80
A411D K424E	-2,579.46
A411E K424D	-2,579.04
R355E K378D	-2,577.06
C391D K424E	-2,576.63

Continued on next page

Table S9 – continued from previous page

Mutation	$\Delta\tilde{E}$
R355D P463E	-2,575.47
K378E K424E	-2,574.65
R355E T430D	-2,574.53
R355E P463D	-2,573.68
R355D T430E	-2,573.40
T385E K424D	-2,573.36
R355D R403E	-2,572.91
R357D K424D	-2,570.56
R355E S514D	-2,570.30
R355E A411D	-2,569.96
G381D K424D	-2,568.07
R355E C391D	-2,567.13
K386E K462P	-2,566.43
R355E K378E	-2,565.15
K386D R408D	-2,564.80
K386E T415D	-2,559.84
R403D K424E	-2,558.39
K424D P463E	-2,556.14
K424D T430E	-2,554.07
R403E K424D	-2,553.58
K386D R408E	-2,549.12
R355E R403D	-2,548.89
A411E K424E	-2,547.16
K386D R454D	-2,542.54
T385E K424E	-2,541.48
R357D K424E	-2,538.68
R355D K462D	-2,538.51
R355E A411E	-2,537.66
G381D K424E	-2,536.19
R355D D405K	-2,534.89
R355D P384E	-2,534.05
R355E T385E	-2,531.98
K386E R408D	-2,531.59
R355E R357D	-2,529.18
R355E G381D	-2,526.69
R355D C391E	-2,526.06
K386D R454E	-2,525.08
R355D L425D	-2,524.30
K424E P463E	-2,524.26
P426D R466E	-2,524.25
K424E T430E	-2,522.19
R403E K424E	-2,521.70
K386D D467E	-2,521.11
K386D P412E	-2,520.13
K424D K462D	-2,519.18
R355D T385D	-2,517.01
K386E R408E	-2,515.91
R355D A522D	-2,515.67
D405K K424D	-2,515.56
R355E P463E	-2,514.76
P384E K424D	-2,514.72
P426D F429D	-2,514.44

Continued on next page

Table S9 – continued from previous page

Mutation	$\Delta\tilde{E}$
R355E T430E	-2,512.69
R355E R403E	-2,512.20
R355D F392D	-2,512.15
F429D R466E	-2,511.19
R355D V503K	-2,510.44
K386E R454D	-2,509.33
C391E K424D	-2,506.73
R355D S514E	-2,506.52
K424D L425D	-2,504.97
R355D L390D	-2,498.09
T385D K424D	-2,497.68
K424D A522D	-2,496.34
R355D Q414D	-2,496.07
R355M K424D	-2,493.64
F392D K424D	-2,492.82
P426D F429E	-2,492.76
K386E R454E	-2,491.87
K424D V503K	-2,491.11
F429E R466E	-2,489.51
K386E D467E	-2,487.90
K424E K462D	-2,487.30
K424D S514E	-2,487.19
K386E P412E	-2,486.92
R355D K462E	-2,486.05
R355D F464E	-2,484.98
R355D Y380E	-2,484.78
D405K K424E	-2,483.68
R355D L425E	-2,482.98
P384E K424E	-2,482.84
P426D R466D	-2,480.00
L390D K424D	-2,478.76
R355E K462D	-2,477.80
Q414D K424D	-2,476.74
C391E K424E	-2,474.85
R355E D405K	-2,474.18
R355E P384E	-2,473.34
K424E L425D	-2,473.09
R355D F464D	-2,472.15
R355D L517E	-2,468.64
G413D P426D	-2,468.53
R355D F515D	-2,468.42
R355D L518D	-2,468.39
F429D R466D	-2,466.94
K386D H519E	-2,466.88
K424D K462E	-2,466.72
T385D K424E	-2,465.80
K424D F464E	-2,465.65
Y380E K424D	-2,465.45
R355E C391E	-2,465.35
G413D R466E	-2,465.28
K424E A522D	-2,464.46
K424D L425E	-2,463.65

Continued on next page

Table S9 – continued from previous page

Mutation	$\Delta\tilde{E}$
R355E L425D	-2,463.59
R355D F377D	-2,463.40
R355M K424E	-2,461.76
R355D Q414E	-2,461.40
F392D K424E	-2,460.94
K424E V503K	-2,459.23
R355E T385D	-2,456.30
G413D F429D	-2,455.47
K424E S514E	-2,455.31
R355E A522D	-2,454.96
R355D P384D	-2,453.96
K424D F464D	-2,452.82
R355E F392D	-2,451.44
R355D T393D	-2,450.37
R355E V503K	-2,449.73
K424D L517E	-2,449.31
K424D F515D	-2,449.09
K424D L518D	-2,449.06
K386D P426E	-2,448.97
L390D K424E	-2,446.88
R355E S514E	-2,445.81
F429E R466D	-2,445.26
Q414D K424E	-2,444.86
F377D K424D	-2,444.07
K386D T415E	-2,443.41
Q414E K424D	-2,442.07
K386D H519D	-2,440.39
G413E P426D	-2,439.39
R355E L390D	-2,437.38
R355D L390E	-2,436.98
G413E R466E	-2,436.14
R355E Q414D	-2,435.36
K424E K462E	-2,434.84
P384D K424D	-2,434.63
G413D F429E	-2,433.79
K424E F464E	-2,433.77
K386E H519E	-2,433.67
Y380E K424E	-2,433.57
K424E L425E	-2,431.77
T393D K424D	-2,431.04
R355D V382E	-2,429.22
R355D Y380D	-2,428.47
G413E F429D	-2,426.33
R355E K462E	-2,425.34
R355E F464E	-2,424.27
R355E Y380E	-2,424.07
R355D C379E	-2,422.37
R355E L425E	-2,422.27
G413D R466D	-2,421.03
K424E F464D	-2,420.94
R355T K424D	-2,420.78
R355C K424D	-2,420.41

Continued on next page

Table S9 – continued from previous page

Mutation	$\Delta\tilde{E}$
R355D A419E	-2,419.67
K386D P412D	-2,417.86
L390E K424D	-2,417.65
K424E L517E	-2,417.43
K424E F515D	-2,417.21
K424E L518D	-2,417.18
R355D V382D	-2,416.95
K386E P426E	-2,415.76
R355D Y423E	-2,415.21
R355D K417D	-2,413.87
R355N K424D	-2,413.54
S383E K386D	-2,412.67
F377D K424E	-2,412.19
R355D G381E	-2,411.65
R355E F464D	-2,411.44
K386E T415E	-2,410.20
Q414E K424E	-2,410.19
V382E K424D	-2,409.89
R355D F515E	-2,409.53
Y380D K424D	-2,409.14
R355E L517E	-2,407.93
R355E F515D	-2,407.71
R355E L518D	-2,407.68
K386E H519D	-2,407.18
R355G K424D	-2,405.73
G413E F429E	-2,404.65
R357E K386D	-2,404.18
C379E K424D	-2,403.04
R355A K424D	-2,402.90
P384D K424E	-2,402.75
R355E F377D	-2,402.69
R355V K424D	-2,402.58
R355D R509D	-2,402.45
R355E Q414E	-2,400.69
A419E K424D	-2,400.34
T393D K424E	-2,399.16
R355D L518E	-2,398.96
R355Q K424D	-2,398.85
R355S K424D	-2,398.19
R355D A522E	-2,397.84
V382D K424D	-2,397.62
P426D K462P	-2,396.02
Y423E K424D	-2,395.88
K417D K424D	-2,394.54
R355E P384D	-2,393.25
K462P R466E	-2,392.77
G381E K424D	-2,392.32
G413E R466D	-2,391.89
R355I K424D	-2,390.58
S383D K386D	-2,390.37
K424D F515E	-2,390.20
R355E T393D	-2,389.66

Continued on next page

Table S9 – continued from previous page

Mutation	$\Delta\tilde{E}$
T415D P426D	-2,389.43
R355H K424D	-2,389.22
R355T K424E	-2,388.90
N331R R355D	-2,388.53
R355C K424E	-2,388.53
T415D R466E	-2,386.18
L390E K424E	-2,385.77
R355D V362D	-2,385.04
K386E P412D	-2,384.65
R355D G416D	-2,383.86
K424D R509D	-2,383.12
F429D K462P	-2,382.96
R355N K424E	-2,381.66
K424D L518E	-2,379.63
S383E K386E	-2,379.46
K386D A419D	-2,379.08
K424D A522E	-2,378.51
V382E K424E	-2,378.01
R355D G431E	-2,377.30
Y380D K424E	-2,377.26
K386D D467K	-2,377.23
R355D Y365D	-2,376.90
T415D F429D	-2,376.37
R355E L390E	-2,376.27
R355D R509E	-2,373.85
R355G K424E	-2,373.85
C379E K424E	-2,371.16
R355A K424E	-2,371.02
R357E K386E	-2,370.97
R355V K424E	-2,370.70
K378D K386D	-2,370.47
N331R K424D	-2,369.20
R355E V382E	-2,368.51
A419E K424E	-2,368.46
K386D T430D	-2,367.94
R355E Y380D	-2,367.76
K386D P463D	-2,367.09
R355Q K424E	-2,366.97
R355D K424I	-2,366.52
R355S K424E	-2,366.31
R355D Y423D	-2,365.83
V382D K424E	-2,365.74
V362D K424D	-2,365.71
G416D K424D	-2,364.53
Y423E K424E	-2,364.00
K386D S514D	-2,363.71
K386D A411D	-2,363.37
R355D F377E	-2,362.94
K417D K424E	-2,362.66
R355D V362E	-2,361.86
R355E C379E	-2,361.66
F429E K462P	-2,361.28

Continued on next page

Table S9 – continued from previous page

Mutation	$\Delta\tilde{E}$
R408D P426D	-2,361.18
K386D C391D	-2,360.54
G381E K424E	-2,360.44
R355E A419E	-2,358.96
R355D N388E	-2,358.89
R355I K424E	-2,358.70
K378E K386D	-2,358.56
K424E F515E	-2,358.32
K424D G431E	-2,357.97
R408D R466E	-2,357.93
Y365D K424D	-2,357.57
R355H K424E	-2,357.34
S383D K386E	-2,357.16
R355E V382D	-2,356.24
R355D V512D	-2,355.02
T415D F429E	-2,354.69
K424D R509E	-2,354.52
R355E Y423E	-2,354.50
R355E K417D	-2,353.16
K424E R509D	-2,351.24
R355E G381E	-2,350.94
R355D G504K	-2,349.18
R355E F515E	-2,348.82
K462P R466D	-2,348.52
R408D F429D	-2,348.12
K424E L518E	-2,347.75
R355D L387D	-2,346.65
K424E A522E	-2,346.63
Y423D K424D	-2,346.50
K386E A419D	-2,345.87
R408E P426D	-2,345.50
K386E D467K	-2,344.02
F377E K424D	-2,343.61
V362E K424D	-2,342.53
K386D R403D	-2,342.30
R408E R466E	-2,342.25
T415D R466D	-2,341.93
R355E R509D	-2,341.74
R355D I358K	-2,339.61
N388E K424D	-2,339.56
P426D R454D	-2,338.92
R355E L518E	-2,338.25
N331R K424E	-2,337.32
K378D K386E	-2,337.26
R355E A522E	-2,337.13
G413D K462P	-2,337.05
R355D R466Y	-2,336.49
K424D V512D	-2,335.69
R454D R466E	-2,335.67
R355D S366D	-2,334.75
K386E T430D	-2,334.73
K386E P463D	-2,333.88

Continued on next page

Table S9 – continued from previous page

Mutation	$\Delta\tilde{E}$
V362D K424E	-2,333.83
G416D K424E	-2,332.65
R408E F429D	-2,332.44
K386D A411E	-2,331.07
K386E S514D	-2,330.50
G413D T415D	-2,330.46
K386E A411D	-2,330.16
K424D G504K	-2,329.85
R355Y K424D	-2,329.44
R355D Y369D	-2,328.48
N331R R355E	-2,327.82
K386E C391D	-2,327.33
L387D K424D	-2,327.32
R355D F429G	-2,327.02
R408D F429E	-2,326.44
K424E G431E	-2,326.09
R355D F429Q	-2,325.87
F429D R454D	-2,325.86
R355D N388D	-2,325.85
Y365D K424E	-2,325.69
T385E K386D	-2,325.39
W353D R355D	-2,325.36
K378E K386E	-2,325.35
R355E V362D	-2,324.33
R355D Q409D	-2,323.39
R355E G416D	-2,323.15
K424E R509E	-2,322.64
R357D K386D	-2,322.59
R355D C432E	-2,321.79
P426D R454E	-2,321.46
I358K K424D	-2,320.28
G381D K386D	-2,320.10
R454E R466E	-2,318.21
P426D D467E	-2,317.49
K424D R466Y	-2,317.16
R355E G431E	-2,316.59
P412E P426D	-2,316.51
R355E Y365D	-2,316.19
S366D K424D	-2,315.42
R355D C379D	-2,315.04
Y423D K424E	-2,314.62
R466E D467E	-2,314.24
R408D R466D	-2,313.68
P412E R466E	-2,313.26
R355D Y421D	-2,313.25
R355E R509E	-2,313.14
R355D R466Q	-2,312.33
F377E K424E	-2,311.73
R408E F429E	-2,310.76
V362E K424E	-2,310.65
R355D F429H	-2,310.62
Y369D K424D	-2,309.15

Continued on next page

Table S9 – continued from previous page

Mutation	$\Delta\tilde{E}$
K386E R403D	-2,309.09
R355D V367D	-2,309.05
F429D R454E	-2,308.40
K386D P463E	-2,308.17
G413E K462P	-2,307.91
K424D F429G	-2,307.69
N388E K424E	-2,307.68
K424D F429Q	-2,306.54
N388D K424D	-2,306.52
K386D T430E	-2,306.10
W353D K424D	-2,306.03
R355E K424I	-2,305.81
K386D R403E	-2,305.61
R355E Y423D	-2,305.12
F429D D467E	-2,304.43
F429E R454D	-2,304.18
Q409D K424D	-2,304.06
K424E V512D	-2,303.81
P412E F429D	-2,303.45
R355D Y369E	-2,302.84
K424D C432E	-2,302.46
R355E F377E	-2,302.23
R408D G413D	-2,302.21
R346D R355D	-2,302.14
G413E T415D	-2,301.32
R355E V362E	-2,301.15
R355D L513D	-2,300.03
R355F K424D	-2,299.59
R355E N388E	-2,298.18
R408E R466D	-2,298.00
K424E G504K	-2,297.97
K386E A411E	-2,297.86
R355Y K424E	-2,297.56
C379D K424D	-2,295.71
L387D K424E	-2,295.44
R355L K424D	-2,295.12
R355E V512D	-2,294.31
Y421D K424D	-2,293.92
K424D R466Q	-2,293.00
T385E K386E	-2,292.18
R355D P521D	-2,291.55
R454D R466D	-2,291.42
K424D F429H	-2,291.29
R355D I410D	-2,290.06
V367D K424D	-2,289.72
R357D K386E	-2,289.38
R355E G504K	-2,288.47
I358K K424E	-2,288.40
G381D K386E	-2,286.89
F429E R454E	-2,286.72
R408E G413D	-2,286.53
R355D T393E	-2,286.10

Continued on next page

Table S9 – continued from previous page

Mutation	$\Delta\tilde{E}$
R355E L387D	-2,285.94
K424E R466Y	-2,285.28
S366D K424E	-2,283.54
Y369E K424D	-2,283.51
R346D K424D	-2,282.81
F429E D467E	-2,282.75
R355D F392E	-2,282.23
P412E F429E	-2,281.77
K424D L513D	-2,280.70
G413D R454D	-2,279.95
R355W K424D	-2,279.86
R355D A363D	-2,278.96
R355E I358K	-2,278.90
Y369D K424E	-2,277.27
K424E F429G	-2,275.81
R355E R466Y	-2,275.78
K386E P463E	-2,274.96
K424E F429Q	-2,274.66
N388D K424E	-2,274.64
C336K R355D	-2,274.16
W353D K424E	-2,274.15
R355E S366D	-2,274.04
R454E R466D	-2,273.96
R408D G413E	-2,273.07
R355D N422D	-2,273.03
K386E T430E	-2,272.89
K386E R403E	-2,272.40
K424D P521D	-2,272.22
Q409D K424E	-2,272.18
R355D I410E	-2,271.34
K386D K462D	-2,271.21
R355D I418E	-2,271.06
I410D K424D	-2,270.73
K424E C432E	-2,270.58
R466D D467E	-2,269.99
R355D K386H	-2,269.41
R355D R408K	-2,269.23
P412E R466D	-2,269.01
R355E Y369D	-2,267.77
R355F K424E	-2,267.71
R355D V407K	-2,267.66
K386D D405K	-2,267.59
P337E R355D	-2,267.52
T393E K424D	-2,266.77
P384E K386D	-2,266.75
R355E F429G	-2,266.31
R355D G431D	-2,265.82
R355E F429Q	-2,265.16
R355E N388D	-2,265.14
R355D R466H	-2,265.08
R355D I418D	-2,265.04
W353D R355E	-2,264.65

Continued on next page

Table S9 – continued from previous page

Mutation	$\Delta\tilde{E}$
R355P K424D	-2,264.51
C379D K424E	-2,263.83
P426D H519E	-2,263.26
R355L K424E	-2,263.24
F392E K424D	-2,262.90
R355E Q409D	-2,262.68
G413D R454E	-2,262.49
Y421D K424E	-2,262.04
C336D R355D	-2,261.92
K424E R466Q	-2,261.12
R355E C432E	-2,261.08
R355D V433D	-2,261.02
R466E H519E	-2,260.01
R355D K417E	-2,259.75
R355D R357K	-2,259.71
A363D K424D	-2,259.63
K424E F429H	-2,259.41
R355D Y365E	-2,259.02
K386D C391E	-2,258.76
G413D D467E	-2,258.52
R355D T393K	-2,258.08
T415D K462P	-2,257.95
R355D P499K	-2,257.84
V367D K424E	-2,257.84
P412E G413D	-2,257.54
R355D R454Q	-2,257.40
R408E G413E	-2,257.39
K386D L425D	-2,257.00
T333D R355D	-2,256.27
C336K K424D	-2,254.83
R355E C379D	-2,254.33
N422D K424D	-2,253.70
R355E Y421D	-2,252.54
R355D V510E	-2,252.46
R355D S366E	-2,252.29
I410E K424D	-2,252.01
R355D F429A	-2,252.01
R355D C391K	-2,251.99
I418E K424D	-2,251.73
Y369E K424E	-2,251.63
R355E R466Q	-2,251.62
R346D K424E	-2,250.93
G413E R454D	-2,250.81
F429D H519E	-2,250.20
K386H K424D	-2,250.08
R355E F429H	-2,249.91
R408K K424D	-2,249.90
T385D K386D	-2,249.71
R355D P491E	-2,248.83
K424E L513D	-2,248.82
K386D A522D	-2,248.37
R355E V367D	-2,248.34

Continued on next page

Table S9 – continued from previous page

Mutation	$\Delta\tilde{E}$
V407K K424D	-2,248.33
P337E K424D	-2,248.19
P337D R355D	-2,248.14
R355W K424E	-2,247.98
R355D F429M	-2,247.09
K424D G431D	-2,246.49
K424D R466H	-2,245.75
I418D K424D	-2,245.71
R355M K386D	-2,245.67
K386D F392D	-2,244.85
R355D K424P	-2,243.96
L335E R355D	-2,243.69
K386D V503K	-2,243.14
V350D R355D	-2,242.98
C336D K424D	-2,242.59
R355E Y369E	-2,242.13
P426E R466E	-2,242.10
K424D V433D	-2,241.69
R346D R355E	-2,241.43
R355D A397K	-2,240.53
K417E K424D	-2,240.42
R357K K424D	-2,240.38
K424E P521D	-2,240.34
T415E P426D	-2,239.79
Y365E K424D	-2,239.69
R355E L513D	-2,239.32
K386D S514E	-2,239.22
I410D K424E	-2,238.85
T393K K424D	-2,238.75
K424D P499K	-2,238.51
R355D F429S	-2,238.13
K424D R454Q	-2,238.07
K386E K462D	-2,238.00
T333D K424D	-2,236.94
P426D H519D	-2,236.77
R355D R454S	-2,236.66
T415E R466E	-2,236.54
R355D S371D	-2,235.13
R346E R355D	-2,235.02
I332E R355D	-2,234.91
T393E K424E	-2,234.89
K386E D405K	-2,234.38
P384E K386E	-2,233.54
R466E H519D	-2,233.52
G413E R454E	-2,233.35
K424D V510E	-2,233.13
S366E K424D	-2,232.96
K424D F429A	-2,232.68
C391K K424D	-2,232.66
R355P K424E	-2,232.63
R355D Y505K	-2,232.32
P426E F429D	-2,232.29

Continued on next page

Table S9 – continued from previous page

Mutation	$\Delta\tilde{E}$
R355D Y495K	-2,232.05
R355D V367E	-2,231.39
E340K R355D	-2,231.25
R355D L387E	-2,231.22
F392E K424E	-2,231.02
R355E P521D	-2,230.84
K386D L390D	-2,230.79
R408D K462P	-2,229.70
K424D P491E	-2,229.50
G413E D467E	-2,229.38
R355E I410D	-2,229.35
P337D K424D	-2,228.81
K386D Q414D	-2,228.77
F429E H519E	-2,228.52
P412E G413E	-2,228.40
K424D F429M	-2,227.76
A363D K424E	-2,227.75
T415E F429D	-2,226.73
K386E C391E	-2,225.55
R355E T393E	-2,225.39
L335E K424D	-2,224.36
R355D V524E	-2,224.02
K386E L425D	-2,223.79
F429D H519D	-2,223.71
V350D K424D	-2,223.65
N331D R355D	-2,223.32
R408D T415D	-2,223.11
C336K K424E	-2,222.95
R355D Q506K	-2,222.35
N422D K424E	-2,221.82
R355E F392E	-2,221.52
R355D R466N	-2,221.50
A397K K424D	-2,221.20
R355D Y489D	-2,220.62
I410E K424E	-2,220.13
I418E K424E	-2,219.85
R355D N450K	-2,219.29
K424D F429S	-2,218.80
K386D K462E	-2,218.75
S349K R355D	-2,218.73
R355E A363D	-2,218.25
K386H K424E	-2,218.20
R355D K386F	-2,218.16
R355D F429W	-2,218.14
R408K K424E	-2,218.02
K386D F464E	-2,217.68
Y380E K386D	-2,217.48
R355D F429N	-2,217.43
R355D R466W	-2,217.43
K424D R454S	-2,217.33
T385D K386E	-2,216.50
V407K K424E	-2,216.45

Continued on next page

Table S9 – continued from previous page

Mutation	$\Delta\tilde{E}$
P337E K424E	-2,216.31
S371D K424D	-2,215.80
R466D H519E	-2,215.76
R346E K424D	-2,215.69
K386D L425E	-2,215.68
I332E K424D	-2,215.58
K386E A522D	-2,215.16
R355D N440K	-2,214.80
T345K R355D	-2,214.74
K424E G431D	-2,214.61
P412D P426D	-2,214.24
R408E K462P	-2,214.02
R355D R466A	-2,213.95
K424E R466H	-2,213.87
I418D K424E	-2,213.83
C336K R355E	-2,213.45
R355D Y421E	-2,213.04
K424D Y505K	-2,212.99
R355D V524D	-2,212.82
K424D Y495K	-2,212.72
R355M K386E	-2,212.46
R355E N422D	-2,212.32
R355D D467Q	-2,212.18
V367E K424D	-2,212.06
E340K K424D	-2,211.92
L387E K424D	-2,211.89
K386E F392D	-2,211.64
R355D A520E	-2,211.48
R355D K386W	-2,211.33
P412D R466E	-2,210.99
R355D Q498K	-2,210.80
C336D K424E	-2,210.71
R355E I410E	-2,210.63
P426E F429E	-2,210.61
R355E I418E	-2,210.35
K386E V503K	-2,209.93
K424E V433D	-2,209.81
R355D F429C	-2,209.77
S383E P426D	-2,209.05
R355E K386H	-2,208.70
K417E K424E	-2,208.54
R355E R408K	-2,208.52
R357K K424E	-2,208.50
R355D P521E	-2,208.20
Y365E K424E	-2,207.81
R454D K462P	-2,207.44
R408E T415D	-2,207.43
R355E V407K	-2,206.95
R355D Y495D	-2,206.89
T393K K424E	-2,206.87
P337E R355E	-2,206.81
K424E P499K	-2,206.63

Continued on next page

Table S9 – continued from previous page

Mutation	$\Delta\tilde{E}$
R355D R466M	-2,206.29
K424E R454Q	-2,206.19
R355D V395E	-2,206.11
R355D A372K	-2,206.04
K386E S514E	-2,206.01
S383E R466E	-2,205.80
R355D F429K	-2,205.54
R355E G431D	-2,205.11
T333D K424E	-2,205.06
T415E F429E	-2,205.05
K386D F464D	-2,204.85
V350E R355D	-2,204.73
K424D V524E	-2,204.69
R355D F377K	-2,204.61
R355E R466H	-2,204.37
R355E I418D	-2,204.33
G413D H519E	-2,204.29
N331D K424D	-2,203.99
R355D V510D	-2,203.30
T333E R355D	-2,203.28
K424D Q506K	-2,203.02
K424D R466N	-2,202.17
F429E H519D	-2,202.03
R355D F429L	-2,201.38
K386D L517E	-2,201.34
K424D Y489D	-2,201.29
K424E V510E	-2,201.25
C336D R355E	-2,201.21
P412D F429D	-2,201.18
K386D F515D	-2,201.12
K386D L518D	-2,201.09
S366E K424E	-2,201.08
T415D R454D	-2,200.85
K424E F429A	-2,200.80
C391K K424E	-2,200.78
R357E P426D	-2,200.56
R355E V433D	-2,200.31
K424D N450K	-2,199.96
S349K K424D	-2,199.40
R355E K417E	-2,199.04
R355E R357K	-2,199.00
R355D Y451K	-2,198.94
K386F K424D	-2,198.83
K424D F429W	-2,198.81
R355E Y365E	-2,198.31
K424D F429N	-2,198.10
K424D R466W	-2,198.10
P426E R466D	-2,197.85
K424E P491E	-2,197.62
K386E L390D	-2,197.58
R355E T393K	-2,197.37
R357E R466E	-2,197.31

Continued on next page

Table S9 – continued from previous page

Mutation	$\Delta\tilde{E}$
R355E P499K	-2,197.13
P337D K424E	-2,196.93
R355E R454Q	-2,196.69
F377D K386D	-2,196.10
S383E F429D	-2,195.99
K424E F429M	-2,195.88
T333D R355E	-2,195.56
K386E Q414D	-2,195.56
K424D N440K	-2,195.47
T345K K424D	-2,195.41
K424D R466A	-2,194.62
R355D N439K	-2,194.32
K386D Q414E	-2,194.10
Y421E K424D	-2,193.71
R355D K386Y	-2,193.59
K424D V524D	-2,193.49
R355D R466S	-2,192.88
K424D D467Q	-2,192.85
L335E K424E	-2,192.48
T415E R466D	-2,192.29
I332D R355D	-2,192.25
K424D A520E	-2,192.15
R355D R454N	-2,192.12
R355D R403K	-2,192.08
K386W K424D	-2,192.00
V350D K424E	-2,191.77
R355E V510E	-2,191.75
R355E S366E	-2,191.58
K424D Q498K	-2,191.47
R355D R466G	-2,191.43
R355E F429A	-2,191.30
R355E C391K	-2,191.28
K424D F429C	-2,190.44
R454E K462P	-2,189.98
R355D V367K	-2,189.90
R355D R466F	-2,189.56
R355D N448K	-2,189.40
A397K K424E	-2,189.32
R466D H519D	-2,189.27
K424D P521E	-2,188.87
R355E P491E	-2,188.12
R355D N360D	-2,187.58
K424D Y495D	-2,187.56
R355D P491D	-2,187.52
R357E F429D	-2,187.50
P337D R355E	-2,187.43
K424D R466M	-2,186.96
K424E F429S	-2,186.92
V395E K424D	-2,186.78
S383D P426D	-2,186.75
A372K K424D	-2,186.71
P384D K386D	-2,186.66

Continued on next page

Table S9 – continued from previous page

Mutation	$\Delta\tilde{E}$
R355E F429M	-2,186.38
G413D P426E	-2,186.38
K424D F429K	-2,186.21
K462P D467E	-2,186.01
K386E K462E	-2,185.54
V341E R355D	-2,185.53
R355D S443K	-2,185.48
K424E R454S	-2,185.45
V350E K424D	-2,185.40
R355D V395K	-2,185.34
F377K K424D	-2,185.28
P412E K462P	-2,185.03
R355D G404K	-2,184.93
K386E F464E	-2,184.47
Y380E K386E	-2,184.27
K424D V510D	-2,183.97
T333E K424D	-2,183.95
S371D K424E	-2,183.92
R346E K424E	-2,183.81
I332E K424E	-2,183.70
R355D D427E	-2,183.54
S383D R466E	-2,183.50
T415D R454E	-2,183.39
R355E K424P	-2,183.25
K386D T393D	-2,183.07
R355D R509K	-2,182.99
L335E R355E	-2,182.98
K386E L425E	-2,182.47
V350D R355E	-2,182.27
K424D F429L	-2,182.05
R355D R454M	-2,181.21
K424E Y505K	-2,181.11
K424E Y495K	-2,180.84
G413D T415E	-2,180.82
V367E K424E	-2,180.18
E340K K424E	-2,180.04
L387E K424E	-2,180.01
R355E A397K	-2,179.82
K424D Y451K	-2,179.61
P412D F429E	-2,179.50
T415D D467E	-2,179.42
R355D Q409E	-2,178.59
P412E T415D	-2,178.44
G413D H519D	-2,177.80
R355E F429S	-2,177.42
R355D V503D	-2,177.20
R355D V503E	-2,176.54
R355E R454S	-2,175.95
A419D P426D	-2,175.46
R355D K424H	-2,175.25
G413E H519E	-2,175.15
K424D N439K	-2,174.99

Continued on next page

Table S9 – continued from previous page

Mutation	$\Delta\tilde{E}$
R355E S371D	-2,174.42
S383E F429E	-2,174.31
R346E R355E	-2,174.31
R355D R466K	-2,174.29
K386Y K424D	-2,174.26
I332E R355E	-2,174.20
R355D I468E	-2,173.69
S383D F429D	-2,173.69
P426D D467K	-2,173.61
K424D R466S	-2,173.55
I332D K424D	-2,172.92
K424E V524E	-2,172.81
R355T K386D	-2,172.81

Table S10: Comparison of ΔE values generated by the GROMOS96-54a7, CHARMM27, and AMBER99 molecular dynamics forcefields. (S) denote stabilizing results, and (D) denote destabilizing results. The magnitude of ΔE values are normalized to assist comparison of results. Mutations below the midpoint line are predicted to destabilize the Spike protein, and mutations above the line are predicted to stabilize the protein structure.

Mutation	ΔE AMBER (kcal/mol)	ΔE CHARMM (kcal/mol)	ΔE GROMOS (kcal/mol)	Norm ΔE AMBER	Norm ΔE CHARMM	Norm ΔE GROMOS	AMBER Norm-Avg
355D	-1757 (S)	-6656 (S)	-1528 (S)	-0.24	-1.24	-0.57	0.448
424D	-1706 (S)	-4543 (S)	-1570 (S)	-0.23	-0.85	-0.59	0.327
355E	-1696 (S)	-6274 (S)	-1546 (S)	-0.23	-1.17	-0.58	0.432
424E	-1674 (S)	-5986 (S)	-1564 (S)	-0.22	-1.12	-0.59	0.418
386D	-1458 (S)	-6057 (S)	-1358 (S)	-0.20	-1.13	-0.51	0.416
386E	-1425 (S)	-5621 (S)	-1612 (S)	-0.19	-1.05	-0.60	0.424
426D	-1288 (S)	-5425 (S)	-1009 (S)	-0.17	-1.01	-0.38	0.349
466E	-1251 (S)	-5981 (S)	-760 (S)	-0.17	-1.12	-0.29	0.355
466D	-1207 (S)	-4549 (S)	-1018 (S)	-0.16	-0.85	-0.38	0.302
415D	-1158 (S)	-5003 (S)	-1127 (S)	-0.15	-0.93	-0.42	0.349
510W	6933 (D)	4600 (D)	2666 (D)	0.93	0.86	1.00	-0.001
382H	6940 (D)	4652 (D)	160 (D)	0.93	0.87	0.06	0.310
382Y	6941 (D)	4671 (D)	8 (D)	0.93	0.87	0.00	0.328
382L	6942 (D)	4656 (D)	-10 (S)	0.93	0.87	0.00	0.331
381H	6974 (D)	4651 (D)	-52 (S)	0.93	0.87	-0.02	0.339
382F	6975 (D)	4693 (D)	2650 (D)	0.93	0.88	0.99	-0.001
381Y	6980 (D)	4645 (D)	2450 (D)	0.93	0.87	0.92	0.027
381W	6983 (D)	4638 (D)	152 (D)	0.93	0.87	0.06	0.315
381F	7021 (D)	4702 (D)	189 (D)	0.94	0.88	0.07	0.310
381R	7474 (D)	5361 (D)	419 (D)	1.00	1.00	0.16	0.281
